# Imidazopyridine hydrazone derivatives exert antiproliferative effect on lung and pancreatic cancer cells and potentially inhibit receptor tyrosine kinases including c-Met

**DOI:** 10.1038/s41598-021-83069-4

**Published:** 2021-02-11

**Authors:** Tahereh Damghani, Fatemeh Moosavi, Mehdi Khoshneviszadeh, Motahareh Mortazavi, Somayeh Pirhadi, Zahra Kayani, Luciano Saso, Najmeh Edraki, Omidreza Firuzi

**Affiliations:** 1 Medicinal and Natural Products Chemistry Research Center, Shiraz University of Medical Sciences, Shiraz, Iran; 2 Department of Medicinal Chemistry, School of Pharmacy, Shiraz University of Medical Sciences, Shiraz, Iran; 3 Department of Physiology and Pharmacology “Vittorio Erspamer”, Sapienza University of Rome, P. le Aldo Moro 5, 00185 Rome, Italy

**Keywords:** Medicinal chemistry, Drug discovery, Drug screening, Medicinal chemistry, Cancer, Cancer models, Cancer therapy, Lung cancer

## Abstract

Aberrant activation of c-Met signalling plays a prominent role in cancer development and progression. A series of 12 imidazo [1,2-α] pyridine derivatives bearing 1,2,3-triazole moiety were designed, synthesized and evaluated for c-Met inhibitory potential and anticancer effect. The inhibitory activity of all synthesized compounds against c-Met kinase was evaluated by a homogeneous time-resolved fluorescence (HTRF) assay at the concentration range of 5–25 µM. Derivatives **6d**, **6e** and **6f** bearing methyl, tertiary butyl and dichloro-phenyl moieties on the triazole ring, respectively, were the compounds with the highest potential. They significantly inhibited c-Met by 55.3, 53.0 and 51.3%, respectively, at the concentration of 25 µM. Synthetic compounds showed antiproliferative effects against lung (EBC-1) and pancreatic cancer cells (AsPc-1, Suit-2 and Mia-PaCa-2) expressing different levels of c-Met, with IC_50_ values as low as 3.0 µM measured by sulforhodamine B assay. Active derivatives significantly blocked c-Met phosphorylation, inhibited cell growth in three-dimensional spheroid cultures and also induced apoptosis as revealed by Annexin V/propidium iodide flow cytometric assay in AsPc-1 cells. They also inhibited PDGFRA and FLT3 at 25 µM among a panel of 16 kinases. Molecular docking and dynamics simulation studies corroborated the experimental findings and revealed possible binding modes of the select derivatives with target receptor tyrosine kinases. The results of this study show that some imidazopyridine derivatives bearing 1,2,3-triazole moiety could be promising molecularly targeted anticancer agents against lung and pancreatic cancers.

## Introduction

Cancer continues to be a major health burden being the first or second common cause of death before the age of 70 in 91 countries and accounting for 9.6 million deaths in 2018 worldwide^1^. Several recent studies have focused on finding new therapies that target specific signalling pathways in cancer cells and in particular on small molecules targeting aberrant kinases^2^. Receptor tyrosine kinases (RTKs) play critical roles in cell proliferation, survival, migration, invasion and other hallmarks of cancer. Aberrant RTKs activation is associated with disease progression in a variety of human malignancies, making them promising drug targets for cancer treatment^3^.

Hepatocyte growth factor receptor or mesenchymal-epithelial transition factor (c-MET) is an important RTK essential for several cellular processes^4,5^. Aberrant activation of hepatocyte growth factor (HGF)/c-Met pathway due to *MET* gene overexpression, amplification, activating mutations, or excessive autocrine or paracrine HGF secretion have been associated with the development of several cancers such as lung, pancreas, gastric, breast, kidney, bladder, ovary, brain and prostate cancers^6–9^.

Over the past few years, different strategies have been pursued to develop HGF/c-Met targeted therapies for management of different types of cancer. Crizotinib was the first small molecule c-Met inhibitor approved in 2011 for treatment of non-small cell lung cancer (NSCLC), which was followed later by approval of cabozantinib for management of metastatic medullary thyroid cancer and very recently capmatinib for treatment of NSCLC^10–12^. In addition, several advanced clinical trials are currently studying the effectiveness of several other HGF/c-Met targeted small molecules and neutralizing antibodies in different types of cancer^13,14^.

**Figure 1:**
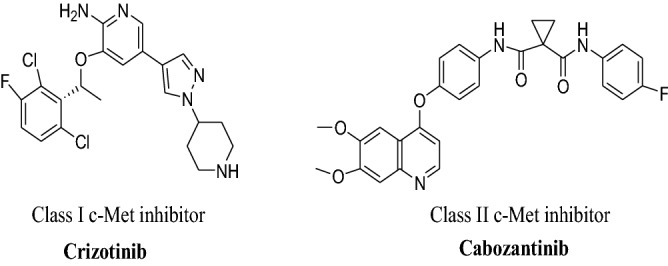
Representative type I and type II c-Met inhibitors.

Small c-Met inhibitors can basically be categorized into two classes (classes I and II) according to their different binding modes with the DFG motif (aspartate-phenylalanine-glycine) of the c-Met activation loop^15^. Class I inhibitors including FDA-approved drugs crizotinib and capmatinib, have a U-shape conformation and bind to the DFG-in conformation, while Class II inhibitors including approved drug cabozantinib, bind to the inactive DFG-out conformation that stretches from the ATP-binding site^16,17^ (Fig. 1).

**Figure 2:**
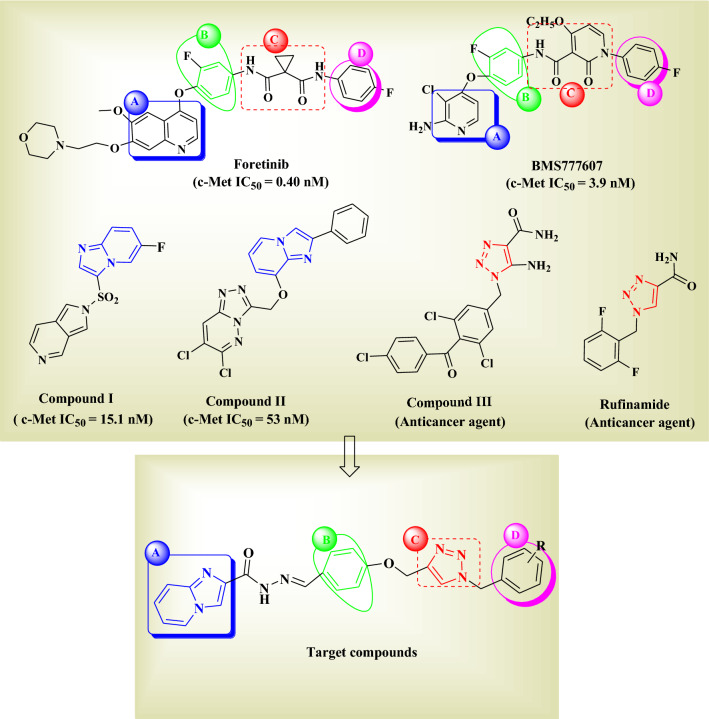
Strategy for the design of imidazo [1,2-α] pyridine derivatives bearing 1,2,3-triazole moiety.

In recent years, studies have shown that class II inhibitors may be more effective than class I inhibitors against the mutations close the active site of c-Met. Moreover, a number of class II c-Met kinase inhibitors have been FDA-approved or have progressed into clinical trials such as foretinib (IC_50_ = 0.40 nM) and BMS777607 (IC_50_ = 3.9 nM)^18,19^ (Fig. 2).

Based on the structural characteristics of type II c-Met inhibitors, we can obtain a general feature which can be divided into four units of A–D^20^ (Fig. 2). Comprehensive structure–activity relationship (SAR) studies about these compounds have suggested that moiety A is usually a fused heterocycle, such as substituted quinoline, substituted pyridine, thieno [2,3-b] pyridine, and pyrrolo [2,3-b] pyridine^21–23^. In addition, B and D are usually a phenyl or substituted phenyl ring in the more promising compounds. As for the moiety C, two different structural properties have been suggested; firstly we should consider that a five-atom linker should be present between moieties B and D. Secondly, the linker should provide hydrogen bond interacting groups and nitrogen atoms for establishing optimal interactions with the active site of c-Met kinase^21,22^. Hence, our strategy became focused on the application of molecular hybridization principle and bioisosteric replacement in order to generate novel type-II c-Met inhibitors (Fig. 2).

Recently, imidazopyridine scaffold such as compounds I (IC_50_ = 15.1 nM) and II (IC_50_ = 53.0 nM) were used as a potent core in many c-Met kinase inhibitors^24,25^. Accordingly, imidazo [1,2-α] pyridine core was employed as the moiety A and a small group like hydrazide was adopted as a linker between A and B parts in order to increase the hydrogen bonding interactions with c-Met active site residues such as Met1160 and Asp1222. Considering part B, the phenyl ring previously reported as optimal group was preserved at this position^26^.

On the other hand, employment of 1,2,3-triazole fragment has been widely popular in the design of anticancer agents such as compounds III and rufinamide (Fig. 2)^27–30^. In this work, 1,2,3-triazole linked to CH_2_–O was employed as the C moiety. Indeed, we assumed that the nitrogen atoms in 1,2,3-triazole and oxygen atom in the linker might serve as potential hydrogen bond acceptor moieties. Consequently, favourable hydrogen bond interactions would be provided with the key residues of c-Met enzyme such as Met1160. Finally, in order to study the effect of various substituents on the c-Met inhibitory activity of the designed structure, different substituted benzyl and heteroaromatic pendants were applied as part D.

Hence, we designed and synthesized a novel series of imidazo [1,2-α] pyridine hydrazone derivatives linked to phenoxy methylene triazole as c-Met kinase inhibitors. The target compounds were evaluated for c-Met kinase inhibitory activity in cell-free and cell-based assays and their antiproliferative effects against different c-Met expressing cancer cell lines were assessed in two- and three-dimensional cell culture models. Moreover, apoptosis induction as well as docking studies were performed for the most promising compounds.

## Results

### Chemistry

**Figure 3:**
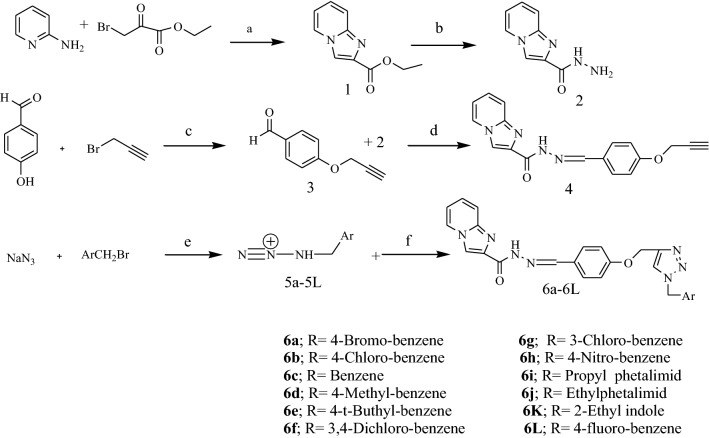
Synthesis procedure: Reagents and conditions were as follows: (**a**) EtOH, 24 h, Reflux. (**b**) NH_2_NH_2_.H_2_O, EtOH, 3 h, Reflux. (**c**) K_2_CO_3_, DMF, 80 °C, 18 h. (**d**) CH_2_Cl_2_, Reflux. (**e**) TEA, THF/water, 70 °C. (**f**) CuSO_4_·5H_2_O (10 mol %) and sodium (2R)-2-[(1S)-12-dihydroxyethyl]-4-hydroxy-5-oxo-2*H*-furan-3-olate (25 mol%), at 40 °C.

The target compounds **6a**–**6l** were synthesized as shown in Fig. 3. Initially, ethyl imidazo [1,2-a]pyridine-2-carboxylate **1** was prepared via the reaction of 2-aminopyridine and ethyl bromopyruvate in refluxing ethanol. In the next step, reaction of compound **1** with hydrazine hydrate under reflux condition gave imidazo [1,2-a] pyridine-2-carbohydrazide **2**. Then, 4-hydroxyaldehyde was reacted with 3-bromoprop-1-yne in the presence of K_2_CO_3_ in DMF at 80 °C in order to produce 4-(prop-2-yn-1-yloxy) benzaldehyde **3**. Further reaction of compound **2** and **3** in EtOH yielded imidazo [1,2-a] pyridine-2-carboxylic acid (4-prop-2-ynyloxy-benzylidene)-hydrazide **4**. Finally, the intermediate compounds **5a**–**l** were prepared via the reaction of different substituted benzyl derivatives with sodium azide in the presence of triethylamine in THF/H_2_O (4:1) solvent system at 70 °C. After around 30 min, intermediate compounds **5a**–**l** were added to the mixture of reaction in the presence of catalytic amount of CuSO_4_·5H_2_O (10 mol%) and sodium (2R)-2-[(1S)-12-dihydroxyethyl]-4-hydroxy-5-oxo-2H-furan-3-olate (25 mol%). The resulting mixture was stirred at 40 °C and completion of the reaction was monitored using thin layer chromatography (TLC) to give compounds **6a**–**6l**.

### Pharmacology

#### c-Met kinase inhibitory effect

Synthesized compounds (**6a**–**6l**) were screened for their c-Met inhibitory activities in vitro by a homogeneous time-resolved fluorescence (HTRF) assay. In this assay, the phosphorylation of a tyrosine kinase (TK) substrate by c-Met kinase is measured by Time Resolved Forster Resonance Energy Transfer (TR-FRET)-based method. Inhibitory activities of the test compounds were measured at three concentrations (5, 10 and 25 μM) as shown in Table 1. It was observed that compounds **6d**, **6e** and **6f** at the concentration of 25 μM significantly inhibited c-Met kinase activity by 55.3, 53.0 and 51.3%, respectively. Derivatives **6h** and **6i** also demonstrated significant inhibitory activities against c-Met enzyme at 25 μM with percent inhibitions of 48.6 and 37.5%, respectively. Derivatives **6k**, **6j**, **6g**, **6l**, **6c** and **6b** did not show any significant inhibition of c-Met kinase. Cabozantinib and crizotinib were also tested as standard Type II and Type I c-Met inhibitors, which showed IC_50_ values of 15.3 and 24.4 nM, respectively.

**Table 1:** c-Met kinase inhibitory activity of synthetic compounds 6a–6l determined by HTRF assay.


Compound	R	% Inhibition		
		5 µM	10 µM	25 µM
**6a**		ND	˂ 10%	36.6 ± 11.1
**6b**		ND	19.2 ± 18.2	33.9 ± 4.2
**6c**		ND	17.9 ± 17.9	27.9 ± 7.3
**6d**		˂ 10%	14.8 ± 13.1	55.3 ± 8.8*
**6e**		19.2 ± 8.6	23.3 ± 4.6	53.0 ± 6.8*
**6f**		38.3 ± 16.0	28.3 ± 19.2	51.3 ± 3.5*
**6g**		ND	˂ 10%	17.2 ± 5.2
**6h**		ND	35.1 ± 9.7	48.6 ± 10.4*
**6i**		ND	ND	37.5 ± 5.4*
**6j**		ND	ND	ND
**6k**		ND	˂ 10%	˂ 10%
**6l**		ND	17.4 ± 19.4	27.7 ± 13.0

*ND* not determined.

*The difference with control was statistically significant (*p* < 0.05). Values are the mean ± S.E.M. of 3–6 separate experiments. Crizotinib and cabozantinib were used as positive controls with IC_50_ values 15.3 nM and 24.4 nM, respectively.

#### Antiproliferative effect against cancer cells

The antiproliferative effects of synthesized derivatives as well as cabozantinib, crizotinib and gemcitabine as reference compounds were investigated in vitro against EBC-1, AsPc-1, Suit-2 and Mia-PaCa-2 cancer cell lines using sulforhodamine (SRB) assay. Incubation of cancer cells with some of the compounds resulted in a dose-dependent antiproliferative effect after 72 h of treatment. Compound **6e** considerably inhibited the proliferation of EBC-1, AsPc-1, Suit-2 and Mia-Paca-2 cells with IC_50_ values of 3.2, 3.1, 3.0 and 15.1 μM, respectively. Moreover, **6d** and **6f** also inhibited the proliferation of cancer cells (Table 2).

**Table 2:** Antiproliferative effects of synthesized compounds assessed by sulforhodamine B (SRB) assay.

Compound	IC_50_ (µM)			
	EBC-1	AsPc-1	Suit-2	Mia-Paca-2
**6a**	> 100	> 100	> 100	> 100
**6b**	> 100	> 100	> 100	60.6 ± 6.9
**6c**	66.7 ± 9.1	> 100	> 100	> 100
**6d**	32.1 ± 4.1	16.7 ± 3.3	41.3 ± 19.5	28.5 ± 1.7
**6e**	3.2 ± 0.4	3.0 ± 0.7	3.9 ± 0.8	15.1 ± 3.2
**6f**	5.1 ± 0.7	20.5 ± 11	86.6 ± 3.6	64.8 ± 4.8
**6g**	> 100	> 100	> 100	> 100
**6h**	60.8 ± 25.5	> 100	> 100	85.2 ± 3.0
**6i**	> 100	> 100	> 100	> 100
**6j**	> 100	> 100	> 100	> 100
**6k**	> 100	> 100	> 100	> 100
**6l**	> 100	> 100	> 100	> 100
**Cabozantinib**	0.059 ± 0.014	1.4 ± 0.1	5.33 ± 1.4	3.8 ± 0.6
**Crizotinib**	0.012 ± 0.006	1.2 ± 0.5	3.6 ± 0.7	1.8 ± 0.7
Gemcitabine*	4.3 ± 3.3	17.2 ± 6.0	3.6 ± 1.9	38.3 ± 22.5

Antiproliferative effect was examined against pancreatic (AsPC-1 and SUIT-2) and lung cancer cells (EBC-1) overexpressing c-MET receptor and pancreatic cancer cells with low MET receptor expression (Mia-Paca-2). IC_50_ values are the mean ± S.E.M. of 3–6 independent experiments.

*Gemcitabine IC_50_ values are expressed in nM.

#### Inhibition of c-Met phosphorylation in cancer cells measured by western blot

**Figure 4:**
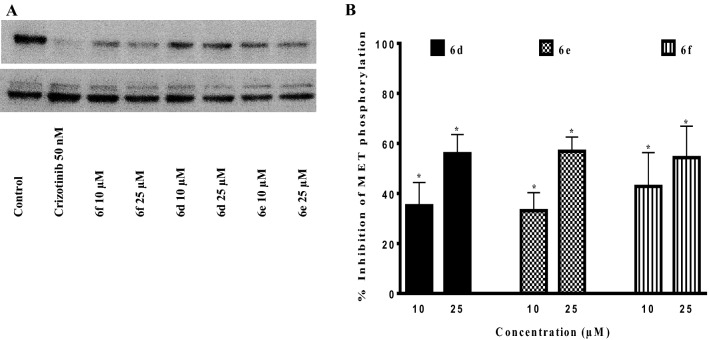
Effect of synthesized derivatives on c-Met phosphorylation in AsPc-1 cancer cells. (**A**) AsPC-1 cells were seeded in 6-well plates and treated with indicated concentrations of synthesized derivatives for 3 h and analyzed by immunoblotting. (**B**) The inhibitory effects of **6f**, **6d** and **6e** compounds were quantified based on alterations of band intensities. *The difference with control was statistically significant (*p* < 0.05). Values represent the mean ± S.E.M. of 3–6 separate experiments.

Compounds **6d**, **6e** and **6f** with highest c-Met inhibitory capacities and antiproliferative effects among the tested derivatives were selected for further evaluations. The inhibitory effects of these derivatives on c-Met phosphorylation were assessed by western blot analysis in AsPc-1 cell. As shown in Fig. 4, treatment with these derivatives at concentrations of 10 and 25 μM for 3 h led to a significant and dose-dependent suppression of c-Met phosphorylation.

#### Inhibition of cancer cell growth in a three-dimensional spheroid model

**Figure 5:**
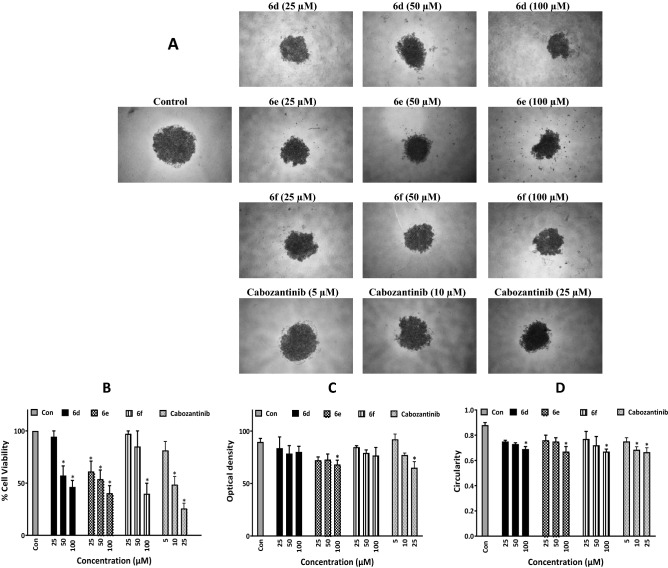
Inhibition of cancer cell growth in three-dimensional spheroid model. Spheroids of AsPc-1 cells were formed by liquid overlay technique in 96-well plates. (**A**) Representative images of spheroids treated with compounds **6d**, **6e** and **6f** at the concentration of 25, 50, and 100 µM are shown**.** The images were prepared with Nikon NIS-Elements imaging software. (**B**) Growth inhibitory effects of compounds on AsPc-1 spheroids was measured by APH assay. (**C**) Optical density and (**D**) Circularity of 3D spheroids after the administrations of synthesized compounds were measured by ImageJ software. The positive controls cabozantinib and gemcitabine had observed IC_50_ values of 8.6 ± 0.7 and 17.8 ± 6.8 µM, respectively. Data are presented as mean ± S.E.M. of at least 3 separate experiments; *The difference with control was statistically significant (*p* < 0.05).

The effect of compounds **6d**, **6e** and **6f** with highest antiproliferative potential among the tested derivatives in monolayer culture, was also tested on cancer cell growth in 3D spheroids. Spheroids were prepared in 96-well plates with liquid overlay technique. A single spheroid of AsPc-1 cells was formed in each well. We observed that 72 h of treatment with all 3 test compounds resulted in a significant dose-dependent effect on spheroids’ growth (Fig. 5). Moreover, structural integrity of spheroids was also quantified and a dose-dependent decrease in circularity and optical density was observed after treatment with all 3 test compounds (Fig. 5).

#### Kinase selectivity profile

The inhibitory activities of the compounds **6d**, **6e** and **6f**, which showed highest c-Met inhibitory potential, were investigated against a panel of 16 human receptor tyrosine kinases using a radiometric assay with ATP concentrations at Km. As shown in Table 3, compounds were not active against most of the RTKs present in kinase panel, which indicates their relative selectivity. However, the derivatives showed inhibitory activities higher than 50% against FMS-like tyrosine kinase-3 (FLT3) and platelet-derived growth factor receptor α (PDGFRA).

**Table 3:** Measurement of the inhibitory effects of synthesized derivatives **6d**, **6e** and **6f** against a panel of different tyrosine kinases.

Kinase	Kinase inhibition (%)^a^		
	**6d**	**6e**	**6f**
ABL1	25	7	31
ALK	17	18	− 17
AXL	16	9	20
KIT	37	10	34
EGFR	14	− 3	17
FGFR1	14	23	24
FLT1	15	0	40
FLT3	71	50	64
KDR	24	19	33
PDGFRA	63	53	71
PDGFRB	14	8	22
RET	− 3	− 19	− 4
RON/MST1R	− 10	− 15	1
ROS1	− 18	14	− 15
NTRK1	52	29	51
NTRK2	34	41	41

^a^Percent inhibition was measured at the concentration of 25 μM.

#### Induction of apoptosis in cancer cells

**Figure 6:**
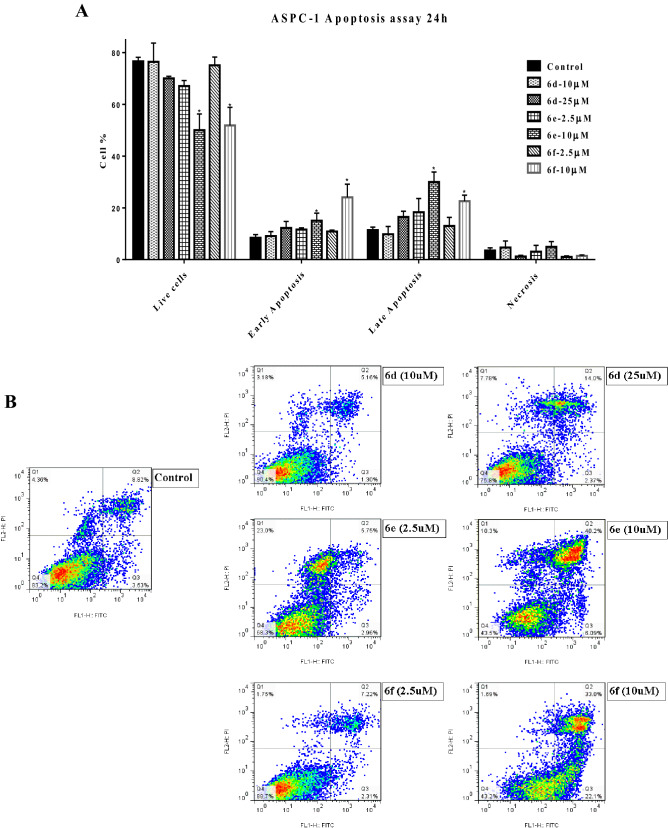
Apoptosis inducing effect of synthesized derivatives on AsPC-1 cancer cells. AsPC-1 cells were treated with compounds **6d** (10 and 25 µM), **6e** (2.5 and 10 µM) and **6f** (2.5 and 10 µM) for 24 h. Apoptosis was determined in cancer cells by annexin V FITC/PI assay and measurements were performed by flow cytometry. (**A**) Each bar represents the average percentage of cells present in each of the 4 quarters ± S.E.M. Data are presented as mean ± S.E.M. of 3 replicates. *Denotes a statistically significant difference between drug-treated cells and untreated control (*p* < 0.05). (**B**) Representative dot plots of AsPC-1 cells treated with different concentrations of synthesized derivatives and monitored by FACS analysis.

A flow cytometric analysis using Annexin V-FITC/PI staining was performed to evaluate the apoptosis induction ability of select compounds on AsPc-1 cell. The results showed that treatment with compounds significantly increased the number of early and late apoptotic cells, while they decreased the number of live cells (Fig. 6).

### In silico studies

#### Molecular docking studies of selected derivatives with c-Met receptor

**Figure 7:**
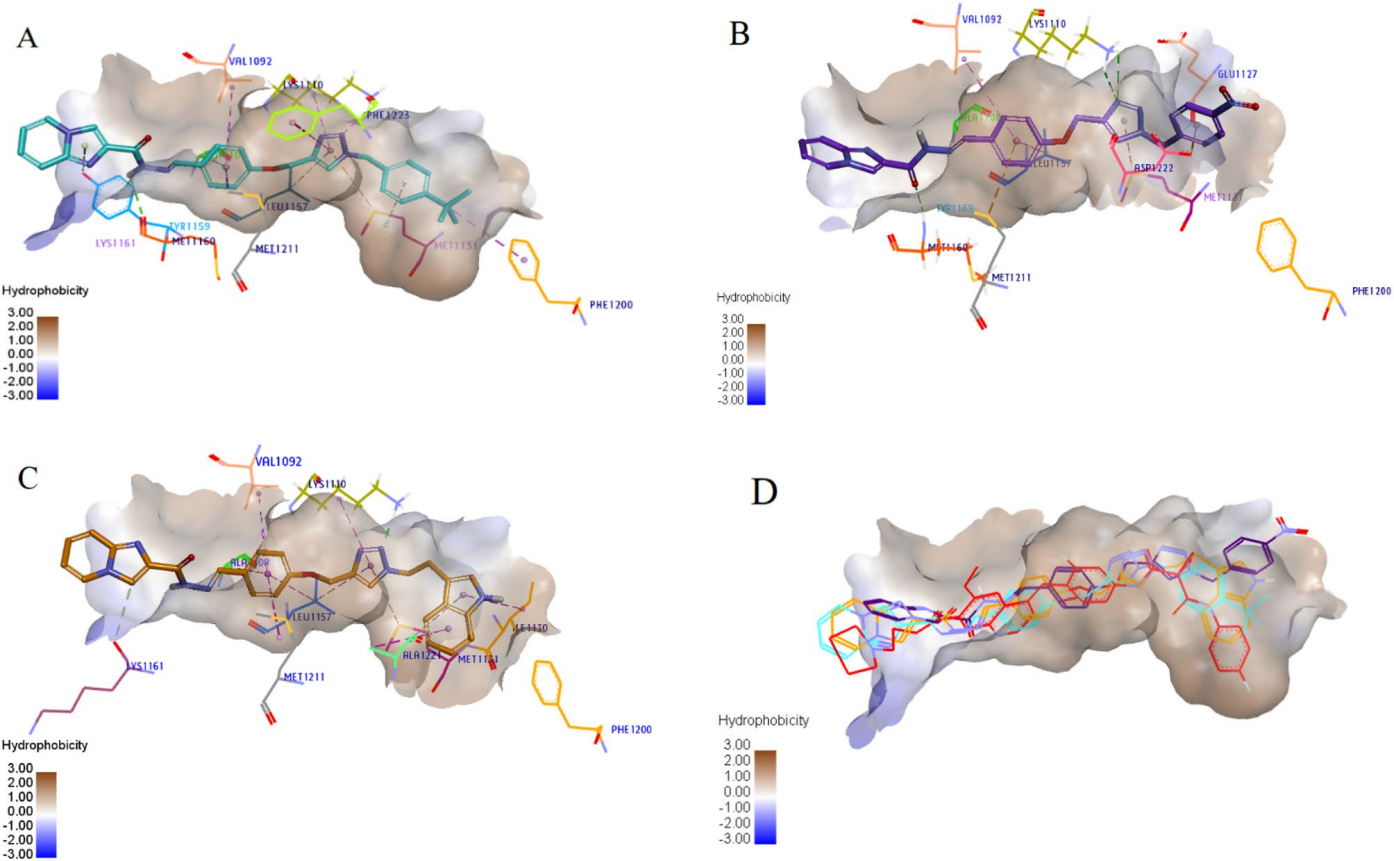
Molecular docking analysis of the interaction of selected synthesized derivatives with c-Met receptor kinase domain. Interactions and binding modes of compounds **6e** (**A**), **6h** (**B**) and **6k** (**C**) are shown. Van der Waals, hydrogen bond, and pi–alkyl interactions were colored as light green, bold green and light pink, respectively. Also, pi–anion, pi–sulfur and pi–pi interactions were colored as dark orange, light orange and dark pink respectively. (**D**) Comparison of binding mode of selected compounds **6e** (blue), **6h** (purple) and **6k** (orange) with the crystallographic ligand foretinib (red) is shown in the active site of c-Met. Images were created by Discovery Studio Client v12.2.0.16349.

Molecular docking analysis was carried out using GOLD 2018 software version 5.6.3^31,32^. The co-crystalized structure of the c-Met (PDB code: 3LQ8) in complex with foretinib was obtained from RCSB Protein Data Bank. The re-docking process of foretinib inside the active site of c-Met kinase was done using two scoring functions of GoldScore and CHEMPLP. The root-mean-square deviation (RMSD) values of re-docking for CHEMPLP and GoldScore were 1.3 Å and 1.55 Å respectively. Therefore, docking analysis was carried out by CHEMPLP fitness function. Interactions of active, intermediate and inactive compounds **6e**, **6h** and **6k** respectively against c-Met ware illustrated in Fig. 7.

Examination of compound **6e** binding mode with c-Met, showed two hydrogen bond interactions with Met1160 and Lys1110. Triazole ring participated in the interactions with Phe1223, Lys1110, Leu1157, and Met1131. The phenyl ring of the methoxy phenyl linker formed pi interactions with Met1211, Val1092 and Ala1108. Of particular interest, the *p*-tertiary butyl benzyl moiety penetrated into the back hydrophobic region and made pi–alkyl interactions with Met1131 (benzyl) and Phe1200 (tertiary butyl). Hence, this compound was able to sterically occupy the back cavity surrounded by Phe1200, Leu1195 and Phe1134 (Fig. 7A). The intermediate activity compound **6h**, showed hydrogen bonds similar to **6e**, however, it missed pi–pi interaction with Phe1223 due to the deflection of 4-nitrobenzene substituent from the hydrophobic cavity (Fig. 7B). The inactive compound **6k**, with less hydrophobic indole substituent lost critical hydrogen bond and pi-pi interactions seen in the more active compounds (Fig. 7C). Furthermore, the binding mode of compounds **6e**, **6h** and **6k** showed that the terminal substituted phenyl group reached to the back hydrophobic pocket similar to foretinib^33^ (Fig. 7D).

#### Molecular dynamics simulation (MDs) of selected derivatives with c-Met receptor

**Figure 8:**
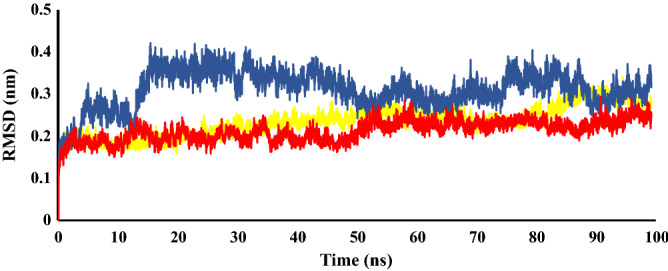
RMSD analysis of MD simulation of the interaction of selected synthesized derivatives with c-Met receptor. To resolve the MD equilibrium time range, the RMSD plot of the protein backbone atoms versus time for the interaction of the active derivatives **6d** (yellow), **6e** (blue), and **6f** (red) with c-Met (PID: 3LQ8) was investigated. The equilibrium time ranges of 44, 15 and 55 ns were observed for complexes of **6d**, **6e** and **6f** with c-Met, respectively. The RMSD figure was drawn by Microsoft Excel 2010.

**Figure 9:**
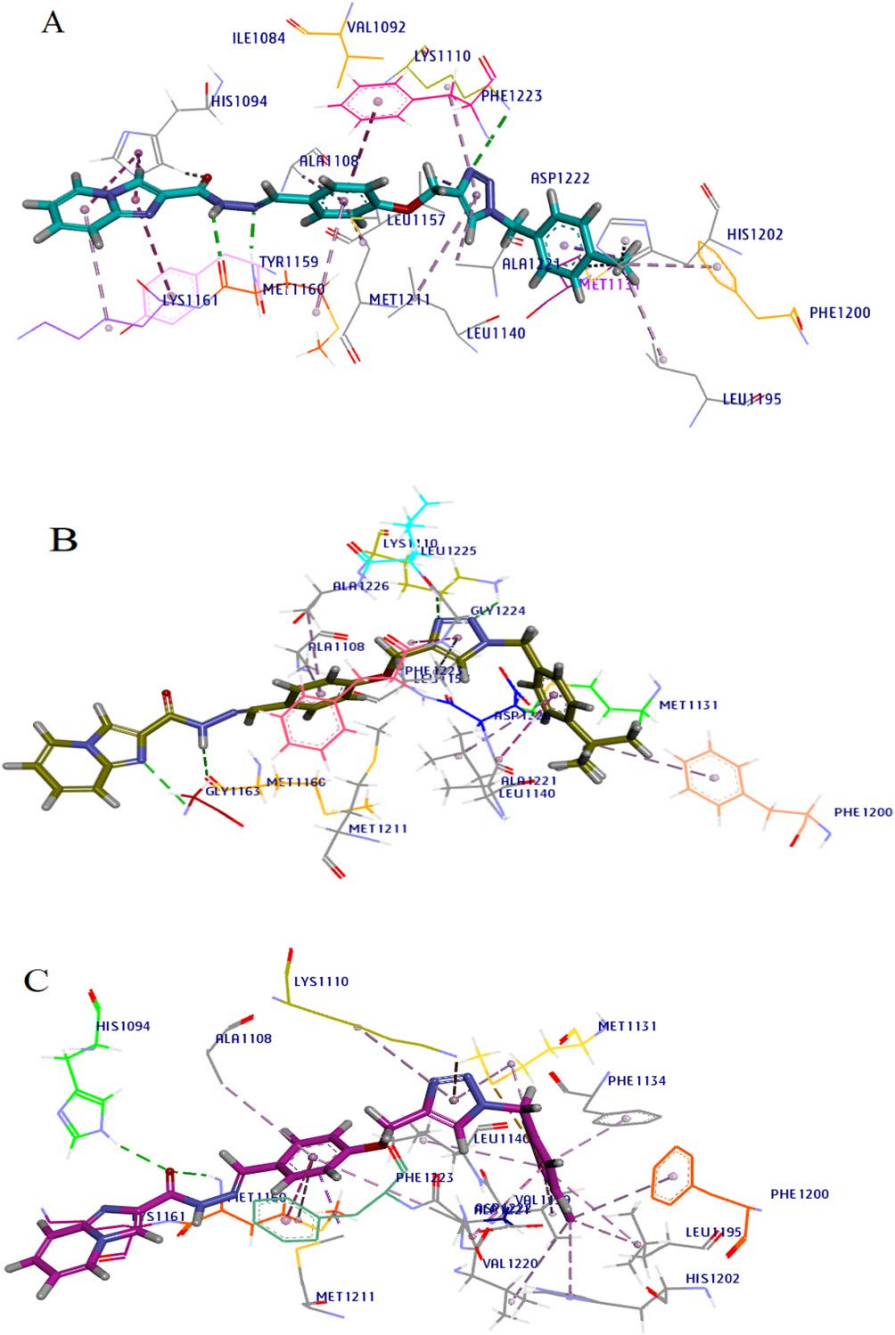
MD simulation analysis of selected compounds against c-Met. 3D MD interaction patterns of compounds **6d** (**A**), **6e** (**B**) and **6f** (**C**) with the highest percent population in cluser1 inside the active site of c-Met kinase are shown. Van der Waals, hydrogen bonds, and pi–alkyl interactions were colored as light green, bold green and light pink, respectively. Furthermore, pi–anion, pi–sulfur and pi–pi interactions were colored as dark orange, light orange and dark pink, respectively. Images were created by Discovery Studio Client v12.2.0.16349.

MD simulation was done for c-Met enzyme in complex with the agents with highest c-Met inhibitory potential among the tested derivatives, **6d**, **6e** and **6f**. The conformational stability of the enzyme was evaluated using calculation of RMSD values of backbone atoms of each frame versus the initial frame against time over the entire course of simulations. The RMSD regular profile was observed about 44 ns for **6d**, 15 ns for **6e** and 55 ns for **6f** complexes (Fig. 8). We calculated the number of hydrogen bonds formed as a function of time with amino acids in the c-Met active site for three complexes during the equilibrium time range in MD simulations. Accordingly, at least one hydrogen bond was made in 96.33% of time for **6d**, 82.11% for **6e** and 97.00% for **6f**. The cluster analysis was done for each complex of compounds **6d**, **6e** and **6f** with c-Met kinase. The percent of population in cluster 1 was 57.00% in compound **6d**, 78.77% in compound **6e** and 84.63% in compound **6f**. Consequently, the representative frame from cluster 1 of each compound was selected for additional analyses (Fig. 9). Compound **6d** shows two critical hydrogen bonds from NH and N-atoms of the hydrazide with Met1160 and a hydrogen bond between triazole ring and Lys1110. Imidazole participated in pi interactions with Tyr1159, His1094 and Lys1161. The phenyl ring of methoxy phenyl linker formed a pi–pi interaction with Phe1223, while the triazole ring made hydrophobic interactions with Ala1221, Leu1140, Lys1110 and Leu1157. Finally, the *p-*methyl benzyl moiety penetrated into the hydrophobic region and made pi interactions to Met1131, Phe1200, His1202, Met1131 and Leu1195. Compound **6e** showed hydrogen bonds to Lys1110, Leu1225, Gly1163 and Met1160. In addition, pi-stacked interactions were made from *p*-tertiary butyl benzyl moiety and triazole ring with Ala1221 and Phe1223, respectively. The *p*-tertiary butyl benzyl moiety also participated in pi–alkyl interactions with Met1131, Leu1140 and Phe1200 (Fig. 9B). The active compound **6f** made two hydrogen bonds to Met1160 and His1094 and a pi–pi interaction to Phe1223. Also, the 3,4*-*dichloro benzyl moiety in the hydrophobic pocket formed several pi interactions from benzene and chloro substituents (Fig. 9C). It was noteworthy that the binding interactions of the **6d**, **6e** and **6f** with c-Met kinase were similar to the co-crystalized foretinib in 3LQ8. There were hydrogen bonds from common substructure of compounds with Met1160 and Lys1110 and pi–pi interaction with Phe1223. Moreover, the phenyl substituents made extra hydrophobic interactions in the back hydrophobic pocket^33^.

#### MM-PBSA analysis

MM-PBSA estimations showed that the native active compounds inside the PDB codes 3LQ8, 4EEV, 5T3Q and 5HTI exhibited the best mean values of average binding energies and ligand binding energy contributions − 185.996 and − 91.670 kJ/mol respectively, see Supplementary Table S1. However, inhibitors **6d**, **6e** and **6f** inside 3LQ8 had the mean values of average binding energy and ligand binding energy contributions of − 170.163, and − 90.430 kJ/mol, respectively.

#### Molecular docking studies of selected derivatives with FLT3 and PDGFRA receptors

**Figure 10:**
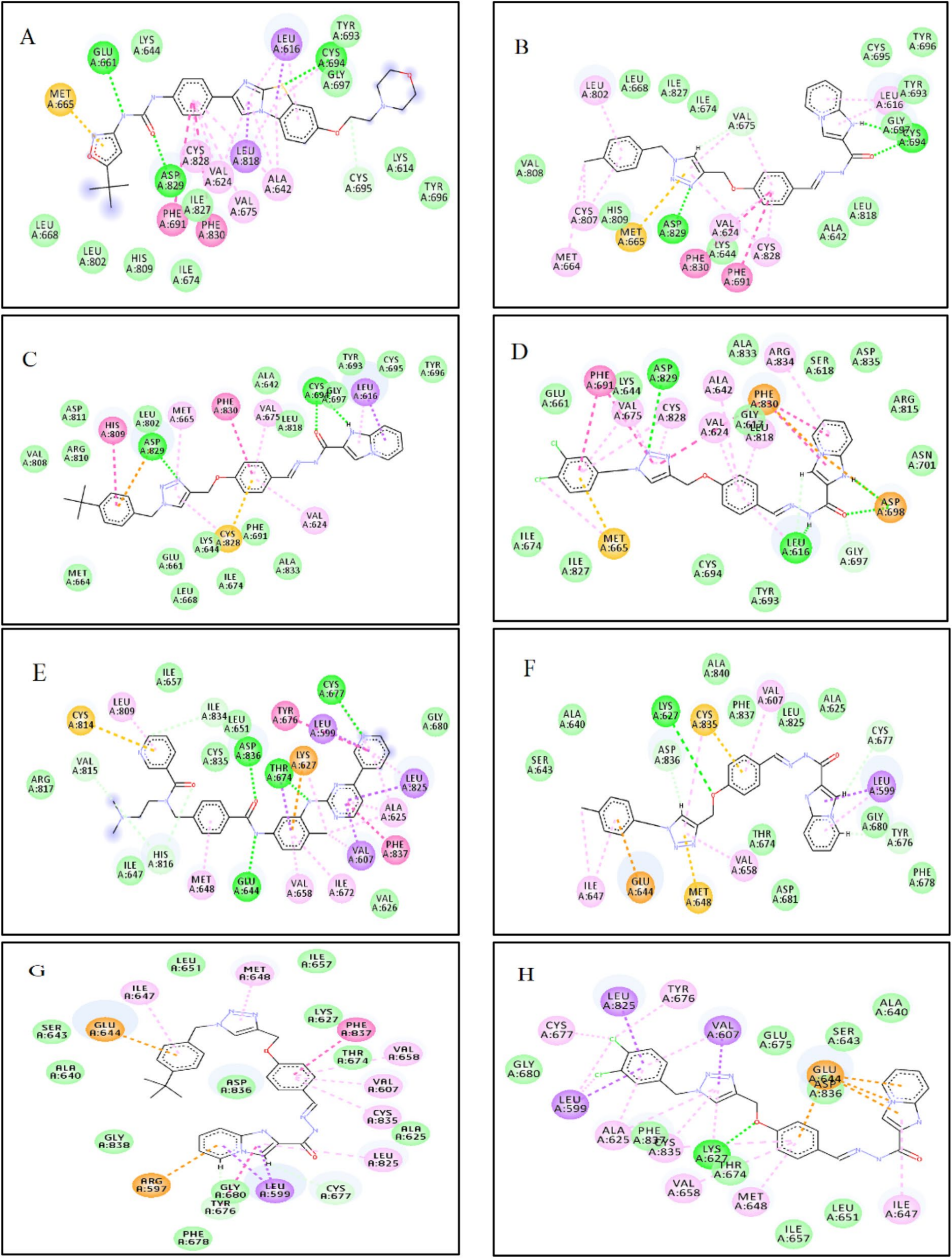
Molecular docking analysis of selected compounds against FLT3 and PDGFRA. 2D Interactions of **P30** (**A**), **6d** (**B**), **6e** (**C**) and **6f** (**D**) inside the FLT3 active site (PDB code 4RT7) and **748** (**E**), **6d** (**F**), **6e** (**G**) and **6f.** (**H**) inside the PDGFRA active site (PDB code 5GRN) are shown. Conventional hydrogen bond, pi–sigma, pi–sulfur, pi–pi, and pi–alkyl interactions were colored as green, purple, orange, dark pink and pink, respectively. Images were created by Discovery Studio Client v12.2.0.16349.

The calculated RMSD values for the re-docking process of the native inhibitors were 0.97 Å and 0.74 Å for FLT3 and PDGFRA, respectively. The docking result of P30 against the active site of FLT3 indicated that there were three key hydrogen bond interactions including Glu661, Asp829 and Cys694 and two pi–pi interactions with Phe830 and Phe691. Compound **6d** showed two hydrogen bonds with Asp829 and Cys694 and the same pi-pi interactions with P30. Derivative **6e** conserved the same hydrogen bonds with **6d** and showed pi-pi interactions with Phe830 and His809. Besides, **6f** illustrated two hydrogen bonds with Asp829 and Leu616 and pi-pi interactions with Phe830 and Phe691 (Fig. 10).

The docking result of compound 748 with the active site of PDGFRA illustrated that there were hydrogen bond interactions with Cys677, Thr674, Glu644 and Asp836 and pi–pi interactions with Tyr676 and Phe837. Compound **6d** had a hydrogen bond with Lys627. Compound **6e** conserved pi–pi interactions with inhibitor 748. Moreover, compound **6f** illustrated a hydrogen bond with Lys627 (Fig. 10).

## Discussion

In this study, 12 novel imidazopyridine hydrazone derivatives linked to phenoxy methylene triazole were synthesized and evaluated for c-Met inhibitory activity in cell free and cell-based assays. The compounds **6d**, **6e** and **6f** bearing methyl, tertiary butyl and dichloro-phenyl moieties on the triazole ring, respectively, in comparison with other synthesized compounds, showed higher c-Met inhibitory potentials, anticancer effects against lung adenocarcinoma and pancreatic ductal adenocarcinoma (PDAC) cells in monolayer and three-dimensional models, inhibited c-Met phosphorylation and also induced apoptosis in cancer cells. Screening of synthesized derivatives against a panel of 16 RTKs showed that derivatives **6d**, **6e** and **6f** may also inhibit FLT3 and PDGFRA enzymes. Computational studies corroborated the experimental findings and showed critical structural features for the interactions between synthesized derivatives and target kinases.

Different lines of evidence in cancer patients as well as preclinical tumor models have shown that HGF/c-Met signaling pathway plays an important oncogenic role in lung cancer^34,35^ as well as in PDAC^36,37^. Therefore, the antiproliferative effects of the compounds were tested against different lung cancer and PDAC cell lines by SRB assay, and it was observed that derivatives **6d**, **6e** and **6f** with the highest c-Met inhibitory potential also suppressed the proliferation of cancer cells. EBC-1 lung cancer cells have *MET* gene amplification and are dependent on c-Met oncogene for proliferation and survival^38^. AsPc-1 and Suit-2 cells also highly express c-Met receptor and their growth is blocked by c-Met inhibitors^39–41^. On the other hand, Mia-Paca-2 cells express very low levels of c-Met protein and are less dependent on this receptor as an oncogenic driver^39,41^. Our findings showed that compounds **6e** and **6f** had low IC_50_ values against EBC-1 cells with the highest level of dependency on c-Met (3.2 and 5.1 µM, respectively), while they were less effective against Mia-Paca-2 cells with lowest level of dependence on c-Met for survival and proliferation (IC_50_ values of 15.1 and 64.8 µM, respectively).

The 3 synthesized derivatives, **6d**, **6e** and **6f**, that exhibited higher c-Met kinase inhibitory potential in HTRF assay in comparison with the other tested derivatives, could also significantly inhibit c-Met phosphorylation in AsPc-1 PDAC cells at 25 µM. Activation of c-Met receptor begins with its phosphorylation on tyrosine 1234 and 1235 residues, which is then followed by the activation of downstream pathways leading to proliferation and survival of cancer cells. We used an antibody that recognizes phosphorylated tyrosine 1234 and 1235 residues, hence the finding that synthesized derivatives block this crucial phosphorylation clearly demonstrates the potential of these compounds in inhibition of important oncogenic pathways.

Assessment of anticancer activity in vitro is usually performed in monolayer cell cultures, however, it is well known that two-dimensional culture systems do not encompass several cellular behaviours that occur in tumors in vivo, including cell–cell interactions, nutrients and oxygen gradients, etc. In this context, three-dimensional spheroid models, which allow cells to mimic some geometry of tumors in vivo and also take into account some important aspects for drug discovery such as drug penetration, may represent more biologically relevant model systems and hence better platforms for preclinical drug screenings^14,42^. Hence, evaluation of the growth-inhibitory effect of compounds **6d**, **6e** and **6f** was performed on AsPc-1 cells grown in 3D cultures. We observed that the synthesized derivatives were able to elicit dose-dependent growth inhibitory effects in AsPc-1 spheroids. These effects could be measured by viability assessments by the acid phosphatase (APH) assay as well as the physical properties of the spheroids including optical density and circularity.

It should be noted that spheroid models are generally much more resistant to different drug therapies as also reported in previous studies^14^. We indeed observed that gemcitabine, which is very effective against AsPc-1 cells in monolayer culture with an IC_50_ value of 17.2 nM, was almost 1000 times less effective against the same cells grown in 3D (IC_50_ = 17.8 ± 6.8 μM). This rather emphasizes the significance of the finding that derivatives **6d**, **6e** and **6f** are able to dose-dependently inhibit spheroid growth. We further observed that when compounds **6d**, **6e** and **6f** were incubated with AsPc-1 cells, the number of early and late apoptotic cells were significantly increased, while the number of live cells were decreased.

The screening of the inhibitory potentials of the most promising derivatives, **6d**, **6e** and **6f**, against a panel of 16 tyrosine kinases, mostly belonging to RTK family, showed that most of the tested tyrosine kinases are not inhibited by the synthesized derivatives. However, compounds exhibited higher than 50% inhibitory effects against FLT3 and PDGFRA at 25 µM. This is generally expected form kinase inhibitors because the kinase domain of RTKs have high levels of similarity and kinase inhibitors designed for a certain RTK may cross-react with other members of the family as well^43^. FLT3 as a key therapeutic target is one of the most commonly dysregulated driver oncogenes in acute myeloid leukemia, and other hematologic malignancies^44,45^. PDGFRA aberrant activation is also correlated with several hallmarks of human cancers including tumor growth, angiogenesis, invasion, and metastasis^46^.

As for the SAR analysis, the obtained results revealed that there is a strong relationship between the nature and position of the phenyl ring substitution and c-Met kinase inhibition. According to the c-Met inhibition results illustrated in Table 1, it could be clearly understood that the lipophilic nature of substituents enhances the inhibitory potency of compounds against c-Met kinase. Compound **6c** bearing benzyl pendant was almost inactive, while, compounds **6d** and **6e** containing *p*-methyl benzyl and *p*-tertiary butyl benzyl pendants, respectively, demonstrated c-Met inhibitory potential of slightly over 50% at 25 µM. Also, c-Met inhibitory effect of compound **6f** substituted with two chlorine groups at *meta*- and *para-*positions of benzyl moiety demonstrated 51.3% inhibitory activity at 25 µM, which was superior to *meta* or *para* chlorinated compounds (**6g** and **6b**).

In addition, the results indicated that the introduction of lipophilic substitutes at the *para* position of the benzyl pendant resulted in higher inhibitory potential compared to the *meta*-substituted compounds. For instance, whereas compound **6b** with chlorine group at C4 position of the benzyl pendant demonstrated 33.9% inhibitory activity at 25 µM, *meta*-chlorinated counterpart **6g**, was almost inactive at this concentration with a percent inhibition of 17.2.

Altogether, it might be concluded that the nature and position of the substituted group on the benzyl ring (part D) have a crucial role on determining of the potency of designed compounds of imidazopyridine scaffold and introducing the lipophilic substituted moieties at *para* position would be beneficial for c-Met kinase inhibitory activity.

In an attempt to further elucidate the binding mode of all compounds, the docking analysis was carried out. The interactions of 3 derivatives with higher (**6e**), intermediate (**6h**) and lower percentages of c-Met inhibition (**6k**), were investigated with the c-Met kinase domain by the docking analysis. The more active compound **6e** made two hydrogen bonds with Met1160 and Lys1110, and a pi–pi interaction with Phe1223 plus several van der Waals interactions. The intermediate activity compound **6h** showed similar hydrogen bonds with **6e**, however, it missed pi–pi interaction with Phe1223 due to the deflection of 4-nitrobenzene substituent from the hydrophobic cavity. In contrast, the inactive compound **6k** showed hydrophobic interactions but lacked some of the critical interactions. Based on the computational analysis, the phenyl group with different lipophilic substituents could occupy hydrophobic back pocket of c-Met kinase domain. Therefore, the higher activity of analogs **6d**, **6e** and **6f** observed in experimental section, might be due to the extra hydrophobic interactions into the hydrophobic back pocket through methyl, tertiary butyl and 3,4 di-chloro benzyl substituents.

In MD simulation studies, number of hydrogen bonds as a function of time was calculated during the equilibrium time ranges and it was found that inhibitors **6d**, **6e** and **6f** formed at least one hydrogen bond during 82% of the equilibrium time. The results showed that the 3 derivatives could form at least one hydrogen bond with 96.33% in **6d**, 82.11% in **6e** and 97.00% in **6f** during the equilibrium time ranges. It was noteworthy that the binding interactions of the **6d**, **6e** and **6f** with c-Met kinase was similar to the co-crystalized foretinib in 3LQ8 and the phenyl substituents made extra hydrophobic interactions in the back hydrophobic pocket.

Moreover, the MM-PBSA analyses showed that complexes of 3LQ8, 4EEV, 5T3Q and 5HTI showed the best mean values of average binding energies and ligand binding energy contributions of − 185.996 and − 91.67 kJ/mol, respectively (Table S1). For compounds **6d**, **6e** and **6e**, these values were less but near to the corresponding average values of potent PDB complexes, which further confirms the experimental results.

Docking analyses of derivatives **6d**, **6e** and **6f** against FLT3 and PDGFRA kinase were also carried out. As shown in Fig. 10, compounds **6d**, **6e** and **6f** contacted with four, three and three residues of FLT3, respectively, via critical hydrogen bond and pi-pi interactions. Moreover, compound **6e** showed a critical pi–pi interaction and compounds **6d** and **6f** showed one hydrogen bond interaction and broad hydrophobic interactions with the active site of PDGFRA.

## Conclusion

In summary, a series of 12 imidazo [1,2-α] pyridine derivatives bearing 1,2,3-triazole moiety was designed, synthesized and evaluated as anticancer agents with c-Met kinase inhibitory potential. Compounds **6d**, **6e** and **6f** showed the highest c-Met inhibitory activities and also blocked the proliferation of EBC-1, AsPc-1, Suit-2 and Mia-PaCa-2 cancer cell lines. Moreover, the western blot analysis of **6d**, **6e** and **6f** illustrated a significant suppression of c-Met phosphorylation. These compounds also dose-dependently inhibited AsPc-1 cells grown in 3D spheroid model and induced apoptosis in the same cells. The screening of these 3 selected compounds for the inhibition of 16 different kinases demonstrated higher than 50% inhibitory effects against FLT3 and PDGFRA. SAR analysis indicated that the steric and lipophilic nature of substituted moieties on the terminal phenyl ring linked to the triazole moiety improves the efficiency of synthesized compounds against c-Met. In this regard, derivatives **6d**, **6e** and **6f** containing 4-methyl, 4-tertiary butyl and 3,4 di-chloro substituents on benzyl pendant, respectively, were the compounds that showed the highest c-Met inhibitory potential among the tested derivatives. Furthermore, in silico evaluation including molecular docking and MD simulation studies of synthetic compounds corroborated the experimental results and demonstrated that three promising compounds **6d**, **6e** and **6f** displayed higher inhibitory potential against c-Met as they could occupy the hydrophobic back pocket of the c-Met. The findings of this study suggest that imidazo [1,2-α] pyridine derivatives bearing 1,2,3-triazole moiety could be potentially interesting molecules meriting further investigation as anticancer agents.

## Methods

### Chemistry

All material and reagents were obtained from the commercial suppliers (Sigma-Aldrich, Fluka and Merck) without further purification. The completion of reactions and purity of the products were checked by TLC on the glass-backed silica gel sheets (Silica Gel 60 GF254) and spots visualized under UV light (254 nm). The melting points of title compounds were measured in open capillary tubes using Thermo Scientific Electrothermal digital apparatus (Thermo Fisher Scientific Inc.). ^1^H NMR (300 or 400 MHz) and ^13^C NMR (75 or 100 MHz) spectra were recorded on a Bruker AV300 or 400 (300 or 400 MHz) spectrometer at ambient temperature and chemical shifts (δ) are expressed in parts per million (ppm) from the solvent resonance (Acetone, CDCl_3_, DMSO-d6). Processing of the spectra was conducted using MestReC (version 4.7.0.0, Mestrelab Research SL, Santiago de Compostela, Spain). Mass spectra were carried out using Agilent 7000 triple quadrupole mass spectrometer at an electron impact mode with an ionization voltage of 70 eV. Elemental analyses for the determination of total C, H and N were performed by Microanalytical Department, Central Laboratories for Research, Shiraz University of Medical Sciences and the results are within 0.4% of the calculated value^47–49^.

Compounds 1–3 were synthesized according to the previously reported procedures^50^.

#### Synthesis of imidazo [1,2-a] pyridine-2-carboxylic acid (4-prop-2-ynyloxy-benzylidene)-hydrazide (4)

A mixture of imidazo [1,2-a] pyridine-2-carbohydrazide (3 mmol, 0.528 g) and 4-(prop-2-yn-1-yloxy) benzaldehyde (3 mmol, 0.480 g) diclorometan (10 ml) was refluxed for 24–48 h. After cooling, the precipitated solid was filtered and washed with diclorometan: n-hexane (2:8) to give pure imidazo [1,2-a] pyridine-2-carboxylic acid (4-prop-2-ynyloxy-benzylidene)-hydrazide white solid Yield : 54%, ^1^H NMR(300 MHz, CDCl_3_):) δ_H_ (ppm): 10.37(s, 1H, C=ONH), 8.30(s, 1H, N=CH), 8.19(m, 2H, Ar–H), 7.79(d, 2H, J = 9 Hz, Ar–H), 7.60(d, 1H, J = 9 Hz, Ar–H), 7.31(overlap with solvent 1H, Ar–H), 7.03(d, 2H, J = 9 Hz, Ar–H), 6.91(t, 1H, J = 7 Hz, Ar–H), 4.75(d, J = 2 Hz, 2H, OCH_2_), 2.57(t, J = 2.1 Hz, H, ≡CH). MS m/z (%):318 (M^+^, 20)^+^, 161 (92), 145 (81), 118 (100), 90 (33), 78(44).

##### Synthesis of substituted derivative of imidazo [1,2-a] pyridine-2-carboxylic acid [4-(1H-[1–3] triazol-4-ylmethoxy)-benzylidene]-hydrazide (**6a**–**6l**)

An aqueous mixture of different derivatives of alkyl or aryl halide (1.1 mmol), sodium azide (1 mmol, 0.065 g) and triethylamine was stirred in THF/water (4:1) for 30 min followed by employment of click reaction in order to synthesize final products **6a**–**6l**^50,51^. To this end, imidazo[1,2-a]pyridine-2-carboxylic acid (4-prop-2-ynyloxy-benzylidene)-hydrazide (4)(1 mmol, 0.318 g) in THF (5 mL), copper sulfate pentahydrate (10 mol%, 0.015 g) and sodium ascorbate (25 mol%, 0.148 g) were added to the aqueous mixture and stirred at 40 °C for 48 h. Upon completion of the reaction (confirmed by TLC), the reaction mixture was dried and the residue was purified by recrystallization from ethyl acetate and n-hexane. All procedure of click chemistry was performed according to previous publications^50,51^.

##### Synthesis of imidazo [1,2-a] pyridine-2-carboxylic acid {4-[1-(4-bromo-benzyl)-1H-[1–3] triazol-4-ylmethoxy]-benzylidene}-hydrazide (**6a**)

Yellow solid; Yield 92%; m.p. 214–218 °C; ^1^H NMR (400 MHz, DMSO‑d6) δ_H_(ppm): 11.57(s, 1H, C=ONH), 8.39(d, 1H, J = 7 Hz, Ar–H), 8.30(m, 2H, Ar–H), 8.09(s, 1H, N=CH), 7.40(m, 3H, Ar–H), 7.34(d, 2H, J = 8 Hz, Ar–H), 7.14(t, 1H, J = 8 Hz, Ar–H), 7.05(d, 2H, J = 8 Hz, Ar–H), 6.88(d, 2H, J = 9 Hz, Ar–H), 6.78(t, 1H, J = 7 Hz, Ar–H), 5.37(s, 2H, CH_2_-O-Ph), 4.98 (s, 2H, N–CH_2_–Ph). ^13^C NMR (100 MHz, DMSO‑d6) δc (ppm): 159.46, 158.36, 147.74, 144.01, 142.75, 138.60, 135.37, 131.68, 130.23, 128.60, 127.70, 127.38, 126.58, 124.85, 121.38, 117.23, 115.70, 115.07, 113.31, 61.15, 52.06. Anal. Calcd for C_25_H_20_BrN_7_O_2_: C 56.61; H 3.80; N 18.49%; found: C 55.57, H 3.54, N 19.48%.

##### Synthesis of imidazo [1,2-a] pyridine-2-carboxylic acid {4-[1-(4-chloro-benzyl)-1H-[1–3] triazol-4-ylmethoxy]-benzylidene}-hydrazide (**6b**)

White solid; Yield 57%; m.p. 200–204 °C; ^1^H NMR (400 MHz, DMSO‑d6) δ_H_(ppm): 11.81(s, 1H, C=ONH), 8.62(d, 1H, J = 7 Hz, Ar–H), 8.54(d, 2H, J = 6 Hz, Ar–H), 8.34(s, 1H, N = CH), 7.65(m, 3H, Ar–H), 7.46(d, 2H J = 9 Hz, Ar–H), 7. 42(t, 1H, J = 7 Hz, Ar–H), 7.36(d, 2H, J = 8 Hz, Ar–H, 7.12(d, 2H, J = 9 Hz, Ar–H), 7.02(t, 1H, J = 7 Hz , Ar–H), 5.64(s, 2H, CH_2_–O–Ph), 5.21 (s, 2H, N–CH_2_–Ph). ^13^C NMR (100 MHz, DMSO‑d6) δc (ppm): 159.46, 158.35, 147.73, 143.90, 142.76, 138.63, 134.96, 132.87, 129.92, 128.75, 128.60, 127.69, 127.38, 126.58, 124.83, 117.23, 115.71, 115.07, 113.32, 61.15, 52.00. Anal. Calcd for C_25_H_20_ClN_7_O_2_: C 61.79; H 4.15; N 20.18%; found: C 59.35, H 3.28, N 20.85%.

##### Synthesis of imidazo [1,2-a] pyridine-2-carboxylic acid [4-(1-benzyl-1H-[1–3] triazol-4-ylmethoxy)-benzylidene]-hydrazide (**6c**)

White solid; Yield 82%; m.p. 215–216 °C; ^1^H NMR (400 MHz, DMSO‑d6) δ_H_(ppm): 11.803(s, 1H, C=ONH), 8.62(d,1H, J = 7 Hz, Ar–H), 8.54(d, 2H, J = 8 Hz, Ar–H), 8.32(s, 1H, N = CH), 7.65(m, 3H, Ar–H), 7.35(m, 6H, Ar–H), 7.12(d, 2H, J = 9 Hz, Ar–H), 7.02(t, 1H, J = 6. Hz, Ar–H), 5.63(s, 2H, CH_2_–O–Ph), 5.21 (s, 2H, N–CH_2_–Ph). ^13^C NMR (100 MHz, DMSO‑d6) δc (ppm):159.47, 158.34, 147.72, 143.90, 142.71, 138.65, 135.97, 128.75, 128.59, 128.14, 127.94, 127.68, 127.38, 126.57, 124.77, 117.23, 115.70, 115.07, 113.31, 61.17, 52.82. MS m/z (%):451 [M+, 13]+, 280 (7), 145 (93), 117 (67), 91 (100). Anal. Calcd for C_25_H_21_N_7_O_2_: C 66.51; H 4.69; N 21.72%; found: C 66.64, H 5.09, N 21.18%.

##### Synthesis of imidazo [1,2-a] pyridine-2-carboxylic acid {4-[1-(4-methyl-benzyl)-1H-[1–3] triazol-4-ylmethoxy]-benzylidene}-hydrazide (**6d**)

White solid; Yield 95%; m.p. 194–200 °C; ^1^H NMR (300 MHz, DMSO‑d6) δ_H_(ppm): 11.81(s, 1H, C=ONH), 8.62(d, 1H, J = 7 Hz, Ar–H), 8.53(d, 2H, J = 4 Hz, Ar–H), 8.29(s, 1H, N = CH), 7.64(m, 3H, Ar–H), 7.37(m, 4H, Ar–H), 7.20(d, 1H J = 7 Hz, Ar–H), 7.12(d, 2H, J = 8 Hz, Ar–H)), 7.02(t, 1H, J = 7, Ar–H), 5.59(s, 2H, CH_2_–O–Ph), 5.20 (s, 2H, N–CH_2_–Ph) 2.50 (overlap with solvent, 3H, CH_3_). ^13^C NMR (75 MHz, DMSO‑d6) δc (ppm): 159.96, 158.83, 148.22, 144.34, 143.17, 139.13, 137.17, 136.46, 129.76, 129.08, 128.43, 128.18, 127.87, 127.07, 125.26, 117.72, 116.18, 115,57, 113.80, 61.67, 53.13, 21.15. Anal. Calcd for C_26_H_23_N_7_O_2_: C 67.08; H 4.98; N 21.06%; found: C 66.56, H 4.59, N 22.72%.

##### Synthesis of imidazo [1, 2-a] pyridine-2-carboxylic acid {4-[1-(4-tert-butyl-benzyl)-1H-[1–3] triazol-4-ylmethoxy]-benzylidene}-hydrazide (**6e**)

White solid; Yield 95%; m.p. 260–264 °C; ^1^H NMR (400 MHz, DMSO‑d6) δ_H_(ppm): 11.81(s, 1H, C=ONH), 8.62(d, 1H, J = 7 Hz, Ar–H), 8.54(d, 2H, J = 8 Hz, Ar–H), 8.30(s, 1H, N = CH), 7.65(m, 3H, Ar–H), 7.38(m, 3H, Ar–H), 7.26(d, 2H, J = 8.Hz, Ar–H), 7.12(d, 2H, J = 9 Hz, Ar–H), 7.02(t, 1H, J = 6 Hz, Ar–H), 5.57(s, 2H, CH_2_–O–Ph), 5.20 (s, 2H, N–CH_2_–Ph) 1.26 (s, 9H, –C (CH_3_)_3_). ^13^C NMR (100 MHz, DMSO‑d6) δc (ppm): 159.46, 158.33, 150.64, 147.70, 143.89, 142.68, 138.65, 133.02, 128.58, 127.75, 127.68, 127.37, 126.57, 125.51, 124.69, 117.23, 115, 70, 115.08, 113.32, 61.63, 52.54, 34.37, 31.01. Anal. Calcd for C_29_H_29_N_7_O_2_: C 68.62; H 5.76; N 19.32%; found: C 68.09, H 5.19, N 20.02%.

##### Synthesis of imidazo [1,2-a] pyridine-2-carboxylic acid {4-[1-(3,4-dichloro-benzyl)-1H-[1–3] triazol-4-ylmethoxy]-benzylidene}-hydrazide (**6f**)

White solid; Yield 68%; m.p. 188–191 °C; ^1^H NMR (400 MHz, DMSO‑d6) δ_H_(ppm): 11.80(s, 1H, C=ONH), 8.62(d,1H, J = 7 Hz, Ar–H), 8.54(d, 2H, J = 8 Hz, Ar–H), 8.36(s, 1H, N=CH), 7.65(m, 5H, Ar–H), 7.38(t, 1H, J = 8 Hz, Ar–H), 7.32(d, 1H, J = 8 Hz, Ar–H), 7.12(d, 2H, J = 9 Hz, Ar–H), 7.02(t, 1H, J = 7 Hz, Ar–H), 5.66(s, 2H, CH_2_–O–Ph), 5.22 (s, 2H, N–CH_2_–Ph). ^13^C NMR (100 MHz, DMSO‑d6) δc (ppm): 159.43, 158.33, 147.71, 143.89, 142.85, 138.65, 136.92, 131.27, 131.01, 130.94, 130.14, 128.59, 128.43, 127.68, 127.41, 126.57, 124.96, 117.23, 115.70, 115.08, 113.31, 61.15, 51.43. Anal. Calcd for C_25_H_19_C_l2_N_7_O_2_: C 57.70; H 3.68; N 18.84%; found: C 57.69, H 3.44, N 18.92%.

##### Synthesis of imidazo [1,2-a] pyridine-2-carboxylic acid {4-[1-(3-chloro-benzyl)-1H-[1–3] triazol-4-ylmethoxy]-benzylidene}-hydrazide (**6g**)

White solid; Yield 73%; m.p. 177–181 °C; ^1^H NMR (400 MHz, DMSO‑d6) δ_H_(ppm): 11.80(s, 1H, C=ONH), 8.62(d, 1H, J = 8 Hz, Ar–H), 8.54(d, 2H, J = 8 Hz, Ar–H), 8.36(s, 1H, N=CH), 7.64(m, 3H, Ar–H), 7.40(m, 4H, Ar–H), 7.28(t, 1H, J = 7 Hz, Ar–H),7.13(d, 2H, J = 9 Hz, Ar–H), 7.02(t, 1H, J = 7 Hz, Ar–H), 5.66(s, 2H, CH_2_–O–Ph), 5.22 (s, 2H, N–CH_2_–Ph). ^13^C NMR (100 MHz, DMSO‑d6) δc (ppm): 159.44, 158.33, 147.72, 143.90, 142.81, 138.65, 138.34, 133.24, 130.70, 128.59, 128.14, 127.84, 127.68, 127.40, 126.66, 126.57, 124.94, 117.23, 115.70, 115.08, 113.32, 61.15, 52.03. Anal. Calcd for C_25_H_20_ClN_7_O_2_: C 61.79; H 4.15; N 20.18%; found: C 60.93, H 4.66, N 21.31%.

##### Synthesis of imidazo [1,2-a] pyridine-2-carboxylic acid {4-[1-(4-nitro-benzyl)-1H-[1–3] triazol-4-ylmethoxy]-benzylidene}-hydrazide (**6h**)

Yellow solid; Yield 98%; m.p. 240–246 °C; ^1^H NMR (300 MHz, DMSO‑d6) δ_H_(ppm): 11.84(s, 1H, C=ONH), 8.62(d, 1H, J = 7 Hz, Ar–H), 8.53(d, 2H, J = 4 Hz, Ar–H), 8.40(s, 1H, N=CH), 8.26(d, 2H, J = 9 Hz, Ar–H), 7.65(d, 2H, J = 9 Hz, Ar–H), 7.54(d, 2H, J = 5 Hz, Ar–H), 7.39(t, 1H, J = 8 Hz, Ar–H), 7.12(d, 2H, J = 9 Hz, Ar–H), 7.02(t, 1H, J = 7 Hz, Ar–H), 5.82(s, 2H, CH_2_–O–Ph), 5.23 (s, 2H, N–CH_2_–Ph). ^13^C NMR (75 MHz, DMSO‑d6) δc (ppm): 159.92, 158.83, 148.18, 147.71, 144.31, 143.86, 143.37, 139.12, 129.54, 129.10, 128.20, 127.90, 127.09, 125.72, 124.43, 117.72, 116.22, 115.56, 113.83, 61.61, 52.40. Anal. Calcd for C_25_H_20_N_8_O_4_: C 60.48; H 4.06; N 22.57%; found: C 59.21, H 4.06, N 23.84%.

##### Synthesis of imidazo [1,2-a] pyridine-2-carboxylic acid (4-{1-[3-(1,3-dioxo-1,3-dihydro-isoindol-2-yl)-propyl]-1H-[1–3] triazol-4-ylmethoxy}-benzylidene)-hydrazide (**6i**)

White solid; Yield 95;%; m.p. 127–135 °C; ^1^H NMR (400 MHz, DMSO‑d6) δH(ppm): 11.85(s, 1H, C=ONH), 8.67(d, 1H, J = 7 Hz, Ar–H), 8.59(d, 2H, J = 9 Hz, Ar–H), 8.31(s, 1H, N=CH), 7.91(m, 4H, Ar–H), 7.70(m, 3H, Ar–H), 7.44(t, 1H, J = 8 Hz, Ar–H), 7.18(d, 2H, J = 9 Hz, Ar–H)), 7.08(t, 1H, J = 7 Hz, Ar–H), 5.25(s, 2H, CH_2_–O–Ph), 4.51(t, 2H, J = 7 Hz, N = N–N–CH_2_–CH_2_), 3.69(t, 2H, J = 7 Hz, CO–N–CH_2_–CH_2_), 2.26(p, 2H, CH_2_–CH_2_–CH_2_). ^13^C NMR (100 MHz, DMSO‑d6) δc (ppm): 167.93, 159.50, 158.33, 147.73, 143.80, 142.24, 138.65, 134.26, 131.74, 128.60, 127.68, 127.36, 126.56, 124.68, 122.96, 117.23, 115.69, 115.06, 113.31, 61.22, 47.26, 34.89, 28.64. Anal. Calcd for C_29_H_24_N_8_O_4_: C, 63.50; H 4.41; N 20.43%; found: C 63.16, H 4.98, N 21.40%.

##### Synthesis of imidazo [1,2-a] pyridine-2-carboxylic acid (4-{1-[2-(1,3-dioxo-1,3-dihydro-isoindol-2-yl)-ethyl]-1H-[1–3] triazol-4-ylmethoxy}-benzylidene)-hydrazide (**6j**)

White solid; Yield 66%; m.p. 254–257 °C; ^1^H NMR (400 MHz, DMSO‑d6) δ_H_(ppm): 11.81(s, 1H, C=ONH), 8.63(d, 1H, J = 7 Hz, Ar–H), 8.54(m, 2H, Ar–H), 8.31(s, 1H, N=CH), 7.85(m, 4H, Ar–H), 7.63(m, 3H, Ar–H), 7.39(t, 1H , J = 8 Hz, Ar–H), 7.08 (d, 2H, J = 9 Hz, Ar–H), 7.01(t, 1H, J = 7 Hz, Ar–H), 5.17(s, 2H, CH_2_–O–Ph), 4.67 (t, 2H, J = 6 Hz, N=N–N–CH_2_–CH_2_), 4.03 (t, 2H, J = 6 Hz, CO–N–CH_2_–CH_2_). ^13^C NMR (100 MHz, DMSO‑d6) δc (ppm): 167.35, 159.41, 158.36, 147.76, 143.90, 142.44, 138.63, 134.50, 131.36, 128.57, 127.69, 127.30, 126.58, 124.97, 123.18, 117.23, 115.70, 115.09, 113.32, 61.18, 47.45, 37.86. Anal. Calcd for C_28_H_22_N8O_4_: C 62.92; H 4.15; N 20.96%; found: C 61.19, H 4.58, N 20.68%.

##### Synthesis of imidazo [1,2-a] pyridine-2-carboxylic acid (4-{1-[2-(1H-indol-3-yl)-ethyl]-1H-[1–3] triazol-4-ylmethoxy}-benzylidene)-hydrazide (**6k**)

White solid; Yield 72%; m.p. 244–246 °C; ^1^H NMR (400 MHz, DMSO‑d6) δ_H_(ppm): 11.81(s, 1H, C=ONH), 10.8(S, 1H, NH) 8.62(d, 1H, J = 7 Hz, Ar–H), 8.55(d, 2H, J = 10 Hz, Ar–H), 8.26(s, 1H, N=CH), 7.65(m, 3H, Ar–H), 7.54(d, 1H, J = 8 Hz, Ar–H), 7.39(d, 1H, J = 7 Hz, Ar–H), 7.35(d, 1H, J = 8 Hz, Ar–H), 7.06(m, 6H, Ar–H), 5.19(s, 2H, CH_2_–O–Ph), 4.66 (t, 2H, J = 7 Hz, N=N–N–CH_2_–CH_2_),3.29 (t, 2H, J = 7 Hz , N = N–N–CH_2_–CH_2_). ^13^C NMR (100 MHz, DMSO‑d6) δc (ppm): 159.49, 158.35, 147.75, 143.90, 142.20, 138.65,136.12, 128.60, 127.69, 127.35, 126.85, 126.57, 124.53, 123.13, 121.05, 118.40, 118.14, 117.23, 115.70, 115.08, 113.32, 111.41, 109.93, 61.26, 50.01, 25.94. Anal. Calcd for C_28_H_24_N_8_O_2_: C 66.65; H 4.79; N 22.21%; found: C 65.51, H 5.00, N 23.08%.

##### Synthesis of imidazo [1,2-a] pyridine-2-carboxylic acid {4-[1-(4-fluoro-benzyl)-1H-[1–3] triazol-4-ylmethoxy]-benzylidene}-hydrazide (**6l**)

White solid; Yield 97%; m.p. 203–205 °C; ^1^H NMR (300 MHz, DMSO‑d6) δ_H_(ppm): 11.84(s, 1H, C=ONH), 8.62(d, 1H, J = 7 Hz, Ar–H), 8.53(d, 2H, J = 4 Hz, Ar–H), 8.33(s, 1H, N=CH), 7.62(d, 3H, J = 9 Hz, Ar–H), 7.47(m, 3H, Ar–H), 7.22(m, 2H, Ar–H), 7.12(d, 2H, J = 9 Hz, Ar–H), 7.02(t, 1H, J = 7 Hz, Ar–H), 5.61(s, 2H, CH_2_–O–Ph), 5.20 (s, 2H, N–CH_2_–Ph). ^13^C NMR (75 MHz, DMSO‑d6) δc (ppm): 159.96, 158.83, 148.18, 144.38, 143.22, 139.11, 132.74, 132.70, 130.87, 130.75, 129.08, 128.18, 127.08, 125.21, 117.72, 116.24, 115.96, 115.54, 113.82, 61.62, 52.49. Anal. Calcd for C_25_H_20_FN_7_O_2_: C 63.96; H 4.29; N 20.88%; found: C 63.81, H 4.77, N 20.17%.

### Pharmacology

#### In vitro enzymatic assays

c-Met kinase inhibitory activity of the test compounds were determined by measuring the phosphorylation level of a biotinylated tyrosine kinase substrate peptide (TK substrate) in a Homogenous Time-Resolved Fluorescence (HTRF) assay. The HTRF KinEASE TK kit was purchased from Cisbio. Optimum substrate, ATP, enzyme concentrations and the enzymatic reaction time were established.

Test compounds were first dissolved in DMSO and then diluted in the reaction buffer containing 50 mM HEPES pH 7.0, 0.1 mM sodium orthovanadate, 0.01% BSA, 0.02% NaN3, 10 mM MgCl_2_, 5 mM MnCl_2_, 2 mM DTT. Four μL of test compound at different concentrations were loaded in a white 384-well plate (Cisbio Cat Number: 6007299), after which 2 μL of c-Met kinase in kinase buffer (0.25 ng/μL) were added to each well. After 10 min of preincubation, the reaction was initiated by adding 2 μL of TK substrate (1 μM final concentration), and 2 μL ATP dissolved in kinase buffer (25 μM final concentration). After 50 min of further incubation at room temperature, the enzyme reaction was terminated by the addition of 10 μL mixed detection solution containing 5 μL Eu^3+^-Cryptate labeled TK antibody in HTRF and 5 μL Steptavidin-XL665 (125 nM final concentration) to allow for detection of the phosphorylated peptide. The Time Resolved-Fluorescence Resonance Energy Transfer (TRFRET) signal was measured after 1 h incubation at room temperature at an excitation wavelength of 337 nm and dual emission detection at 665 and 620 nm with a Bio-Tek multimode plate reader (Model Cytation 3).

The inhibition rate (%) was calculated using the following equations:

$$ {\text{Ratio}}_{{{665}/{62}0}} = {\text{ Emission}}_{{\text{665 nm}}} /{\text{Emission}}_{{{62}0{\text{ nm}}}} , $$

$$ \Delta {\text{R }} = \, \left( {{\text{Ratio sample}}_{{{665}/{62}0}} - {\text{ Ratio background}}_{{{665}/{62}0}} } \right) \times {1}00/{\text{ Ratio background}}_{{{665}/{62}0}} , $$

$$ {\text{Inhibition }}\left( \% \right) \, = \, \left( {\Delta {\text{R}}_{{{\text{Control}}}} - \, \Delta {\text{R}}_{{{\text{Sample}}}} } \right) \times {1}00 \, / \, \Delta {\text{R}}_{{{\text{Control}}}} . $$

Background samples contained all reagents except for the enzyme. Control wells contained the same amount of DMSO contained in sample. The maximum level of DMSO did not exceed 2%. Kinase inhibition profile was examined by Kinase Radiometric Assays with ATP concentration at Km for 16 kinases.

#### Cell culture

EBC-1 (human lung adenocarcinoma cells), Suit-2 and Mia-Paca-2 cells (human PDAC cells) were obtained from Japanese Collection of Research Bio Resources Cell Bank (JCRB). AsPc-1 (human PDAC cells) were obtained from Iranian Biological Resource Centre, Tehran, Iran. EBC-1, AsPc-1 and Suit-2 cells were cultured in RPMI 1640 medium containing 10% heat-inactivated fetal bovine serum (FBS) and 1% penicillin/streptomycin. Mia-Paca-2 cells were grown in DMEM low glucose, containing 10% heat-inactivated FBS and 100 U/mL penicillin/streptomycin. All cells were grown in monolayer culture at 37 °C in a humidified incubator with 5% CO_2_.

#### Assessment of the antiproliferative effect by sulforhodamine B (SRB) assay

Antiproliferative activities of synthetic compounds were evaluated by SRB assay as previously described^14^. Synthetic compounds were first dissolved in DMSO and then diluted in growth medium. The cells were trypsinized and a homogenous cell suspension was prepared. One-hundred µl of cell suspension at a density of 5 × 10^4^ cells/mL were seeded in 96-well flat bottom plates. After 24 h of incubation to allow cells to attach and resume optimal growth, 100 µL of synthesized derivatives were added at different concentrations in triplicate and incubated for an additional 72 h at 37 °C. The maximum concentration of DMSO in each well did not exceed 0.5%. The cells were then fixed by gentle addition of 50 μL cold trichloroacetic acid (TCA) 50% (w/v, 10% final concentration) and incubated for 60 min at 4 °C. The supernatant was discarded, and the plates were washed four times with distilled water and air dried. Afterwards, 100 μL of SRB solution 0.04% (w/v) dissolved in 1% acetic acid was added to each well, and plates were incubated for 15 min at room temperature. After staining, the unbound dye was removed by washing four times with 1% acetic acid and the plates were then air dried. Bound stain was subsequently solubilized with 150 μL of 10 mM Tris base solution, and the absorbance was recorded at a wavelength of 540 nm with a Bio-Tek microplate reader (Model Synergy HTX).

#### Measurement of c-Met phosphorylation in cancer cells by western blotting

c-Met phosphorylation in AsPc-1 pancreatic ductal adenocarcinoma cells was measured by immunoblotting. AsPc-1 cells were seeded in 6-well plates at a density of 250,000 cells/mL and incubated at 37 °C for 24 h. The synthetic compounds at different concentrations were introduced into the well and incubated for 3 h. The cells were then harvested in ice-cold RIPA lysis buffer (20 mM Tris base, 150 mM NaCl, 1% Np40, 1 mM EDTA, 5% sodium deoxycholate and 0.1% SDS, pH 8.0) containing phenylmethylsulfonyl fluoride (PMSF) 1 µM, Na_4_O_7_P_2_ 10 mM and Na_3_VO_4_ 2 mM, by use of scrapers. PMSF and a protease inhibitor cocktail (Roche) were added to the extraction buffer in order to prevent the breakdown of the proteins. The lysates were vortex mixed for 20 min and then centrifuged at 12,000×*g* for 20 min at 4 °C. Supernatants were collected, transferred into fresh tubes and stored at − 20 °C until use. Protein contents of the cell extracts were determined by a bicinchoninic acid protein assay kit (Quanti-Pro BCA, Sigma-Aldrich, St. Louis, USA) using bovine serum albumin as the protein standard. Equal amounts of extracted protein were separated on 7.5% SDS-PAGE and transferred onto the PVDF membrane at 150 V in 1 h. Nonspecific binding sites on the membranes were blocked with 4% BSA dissolved in Tris buffer saline containing 0.1% Tween-20 (TBST) for 50 min at room temperature. Proteins were then detected by specific primary antibodies, rabbit monoclonal anti-p-MET (dilution 1:1000, catalogue number: 3126, Cell Signaling, Danvers, MA) and rabbit monoclonal anti-MET (dilution 1:1000, catalogue number: 4560, Cell Signaling, Danvers, MA) overnight at 4 °C. After incubation with secondary antibody (goat anti-rabbit horse radish peroxidase conjugated IgG, Cell Signaling, Danvers, MA) for 1 h at room temperature, immune-reactive bands were visualized using enhanced chemiluminescence detection substrates (Thermo Fisher Scientific, Waltham, MA). Images were obtained with a G: Box Chemi-XR5 GeneSys image analyzer. The bands intensities were calculated with the software Gene Tools (SyneGene, Cambridge, UK) for Windows.

#### Measurement of the anticancer effect in three-dimensional spheroid assay

Three-dimensional spheroid cell cultures were performed with liquid overlay method. Agarose 1.5% dissolved in RPMI was used to coat 96-well flat bottom plates as follow. Agarose powder was dissolved in RPMI (1.5%) and sterilized in an autoclave. Then, 50 µL of agarose solution was pipetted into each well and was left to solidify at room temperature for at least 2 h.

A suspension of AsPc-1 cells in RPMI medium containing FBS 10% at a density of 2 × 10^5^ cells/mL was prepared and 125 µL of this cell suspension was added to each well. Then the plates were centrifuged at 700×*g* for 5 min and incubated under standard culture conditions for 48 h, which allowed one spheroid to form in each well. Afterwards, 100 µL of the medium was removed from each well and spheroids were treated with synthesized derivatives diluted in fresh medium containing 10% FBS.

After 72 h of drug treatment, cell viability was measured by the APH assay, which is based on the hydrolysis of the p-nitrophenyl phosphate (pNPP) by intracellular acid phosphatases present in viable cells and its conversion to yellow p-nitrophenol. Then 160 µL of the medium was removed and 200 µL of APH solution containing 2 mg/mL pNPP dissolved in 0.1 M sodium acetate at pH 4.8 were added to each well and incubate for 120 min at 37 °C in an incubator.

After 120 min of incubation, the reaction was stopped by the addition of 10 µL NaOH 1 M and the absorbance was recorded at 405 nm within 10 min by a Bio-Tek microplate reader (Model Synergy HTX). The images of spheroids were prepared with Nikon NIS-Elements AR imaging software for Windows version 4.30.01.

#### Assessment of apoptosis induction in cancer cells

Apoptosis was evaluated by FACS analysis with Annexin V-FITC/PI staining kit (BD Pharmingen, San Diego, CA, USA). AsPc-1 cells were seeded in 6-well plates at a density of 1 × 10^5^ cells/mL. After 48 h, the synthesized derivatives at different concentrations were added and incubated for 24 h. The cells were then harvested by adding trypsin 0.1%, transferred to 1.5 mL tubes and washed twice with PBS. The cells were finally stained with Annexin V-FITC (5 µL) and propidium iodide (5 µL), followed by analysis by a FACS Calibur flow cytometer (Becton Dickinson, Mountain View, CA, USA). The drug effect on the apoptosis rate was evaluated based on the fluorescence signal of 10,000 events. The results were analyzed using FlowJo software version 10.1^52^.

### In silico studies

#### Molecular docking

Molecular docking analysis was carried out to investigate the binding modes of the key molecular interactions between the synthetic compounds with highest anticancer activities and the binding site of c-Met kinase using GOLD 2018 software version 5.6.3^31,32^. The binding site was considered 8 Å around the foretinib in the crystallographic structure of c-Met. For all docking runs, the genetic algorithm parameters were set as default values: a population size of 100, a gene mutation frequency of 95, a crossover frequency of 95 and number of operations of 100,000. Discovery Studio Client v12.2.0.16349 was used to analyze the docking results and create the images of interaction patterns^53^. All compounds were sketched using Marvin Sketch 18.20.0^54^ and energy of molecules were optimized with Open Babel 2.4.0 using steepest descent algorithm^55^. The preparation of protein structure and the addition of all hydrogens were done by Discovery Studio Client v12.2.0.16349. Also, definition of binding site of the enzyme for docking was done automatically by the coordinates of the native ligand foretinib. The RMSD value of redocking process of foretinib inside 3LQ8 with two scoring functions in GOLD namely CHEMPLP, and GoldScore were calculated to validate and find the most appropriate scoring function for thr docking analyses.

Since the results of the kinase panel screening showed that compounds **6d**, **6e** and **6f** are also able to inhibit FLT3 and PDGFRA receptors, we performed docking analysis to better understand the nature of the interaction of these derivatives with the two RTKs. The 3D structures of the FLT3 (PID: 4RT7) and PDGFRA (PID: 5GRN) were obtained from RCSB Protein Data Bank. Compounds P30 and 748 were re-docked inside the active sites of FLT3 and PDGFRA, respectively, using CHEMPLP scoring functions in the GOLD software.

#### Molecular dynamics simulation

MD simulation was done applying the Gromacs 2019.1 simulation package on a Linux GPU server for c-Met enzyme in complex with compounds **6d**, **6e** and **6f**. The MD simulations were run using Amber99sb force filed at neutral pH 7.0 and the mean temperature of 300 K. The calculation of AM1 partial charges was done by Chimera software and the creation of force filed parameters was accomplished by acpype as previously reported^56^. Then, a dodecahedral solute-box was defined and further TIP3P water molecules filled the box completely. To make the system neutral, 0.15 mol/L sodium chloride was added to replace adequate number of water molecules. The use of the steepest descent algorithm through a 100 ps run was done in order to minimize the system. Afterwards, a force constant of 1000 kJ mol^−1^ nm^−2^ was performed to restrain the atom locations of the macromolecule and ligand. Then, 500 ps NVT simulation was run and the regulation of the temperature was done via V-rescale thermostat to 300 K. Then, for the NPT step, the system pressure was set to 1 bar during the 500 ps equilibration phase. The production MD was run for 100 ns simulation time under a well equilibrated system. Two methods of particle-mesh Ewald (PME) and LINCS constraint were used to investigate the long-ranged electrostatic contributions and restrain the length of all covalent bonds respectively. At the end, the protein complex was centred back in the box and the trajectory was amended based on the periodic boundary condition. The RMSD Å of the protein backbone atoms of each frame against the initial frame as the reference was calculated to define the equilibrium time period. The RMSD figures were drawn by Microsoft Excel 2010 for Windows. Also, the clustering method was applied in the equilibrium time range by gromos approach and cut off value 0.11 to extract the representative frames of the simulation as also reported previously^23^.

#### Binding free energy calculations using MM-PBSA

The binding free energy of four PDB codes of 3LQ8, 4EEV, 5T3Q and 5HTI in complex with potent inhibitors foretinib, LY2801653, 75H and 66L respectively, plus three complexes with **6d**, **6e** and **6f** was calculated using MM-PBSA approach. MM-PBSA analyses were performed using the g_mmpbsa tool provided by Kumari and colleagues^57,58^. MM-PBSA estimates the binding free energies by means of combination of molecular mechanics and continuum solvent models. Other than calculation of binding energy components, it can also report the individual energy contributions of amino acids. In this study, the equilibrium time range elucidated by RMSD graph of all complexes was applied for precise MM-PBSA estimation. The adaptive Poisson-Boltzmann Solver (APBS) approach calculated the electrostatic energy, VDW energy and polar solvation energy contributions while the non-polar contributions of solvation energy were estimated by the Solvent-accessible surface area (SASA) approach. Grid spacing of 0.5 Å and probe radius of 1.4 Å were used for SASA estimate with solvent dielectric constant value of 80, and solute dielectric constant value of 2. Average binding energy of each complex and energy contribution of ligands were specified at the end.

#### Statistical analysis

All data were expressed as the mean ± S.E.M. of 3–6 repeated experiments. Statistical significance of the differences was determined by one-way ANOVA with Tukey Post Hoc test.

## Supplementary Information

Supplementary Information 1.

Supplementary Information 2.
