# Supplementary material for: Imidazopyridine hydrazone derivatives exert antiproliferative effect on lung and pancreatic cancer cells and potentially inhibit receptor tyrosine kinases including c-Met
Source: Sci Rep. 2021 Feb 11;11:3644. doi: 10.1038/s41598-021-83069-4 (PMC7878917; doi:10.1038/s41598-021-83069-4)
Supplement: Supplementary file 1 — Supplementary Information 1. [file 41598_2021_83069_MOESM1_ESM.docx]

**Supplementary Data**

**Imidazopyridine hydrazone derivatives exert antiproliferative effect on lung and pancreatic cancer cells and potentially inhibit receptor tyrosine kinases including c-Met**

Tahereh Damghani^a,b^, Fatemeh Moosavi^a^, Mehdi Khoshneviszadeh^a,b^, Motahareh Mortazavi^a^, Somayeh Pirhadi^a^, Zahra Kayani^a^, Luciano Saso^c^, Najmeh Edraki^a*^, Omidreza Firuzi^a*^

*^a^ Medicinal and Natural Products Chemistry Research Center, Shiraz University of Medical Sciences, Shiraz, Iran.*

*^b^ Department of Medicinal Chemistry, School of Pharmacy, Shiraz University of Medical Sciences, Shiraz, Iran*

*^c^ Department of Physiology and Pharmacology "Vittorio Erspamer", Sapienza University of Rome, P. le Aldo Moro 5, 00185 Rome, Italy*

**Corresponding authors:**

Omidreza Firuzi, MD PhD

Medicinal and Natural Products Chemistry Research Center

Shiraz University of Medical Sciences, Shiraz, Iran

Phone: (+98)-71-3230-3872

Email: [firuzio@sums.ac.ir](mailto:firuzio@sums.ac.ir)

Najmeh Edraki, PhD

Medicinal and Natural Products Chemistry Research Center

Shiraz University of Medical Sciences

Shiraz, Iran

Phone: (+98)-71-3230-7869

Email: [edrakin@sums.ac.ir](mailto:edrakin@sums.ac.ir)

**^1^H-NMR and ^13^C-NMR Spectrum of synthetic compounds (6a-6l).** Processing of the spectra was conducted using MestReC (version 4.7.0.0, Mestrelab Research SL, Santiago de Compostela, Spain):


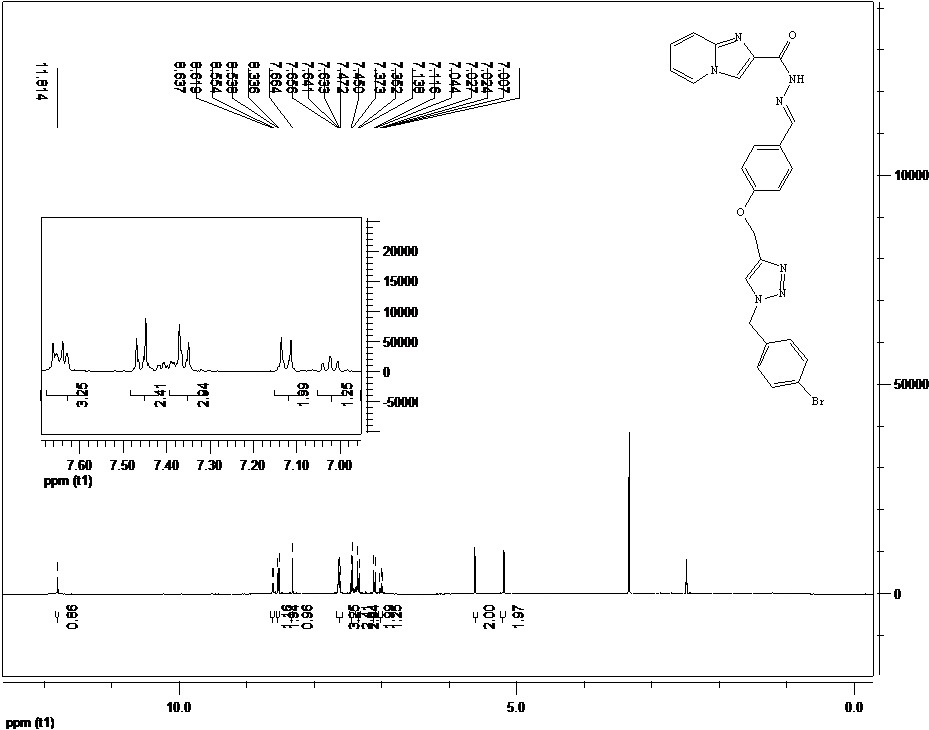


**Fig. S1.** ^1^H NMR spectrum of imidazo [1,2-a]pyridine-2-carboxylic acid {4-[1-(4-bromo-benzyl)-1H-[1,2,3]triazol-4-ylmethoxy]-benzylidene}-hydrazide **(6a)**


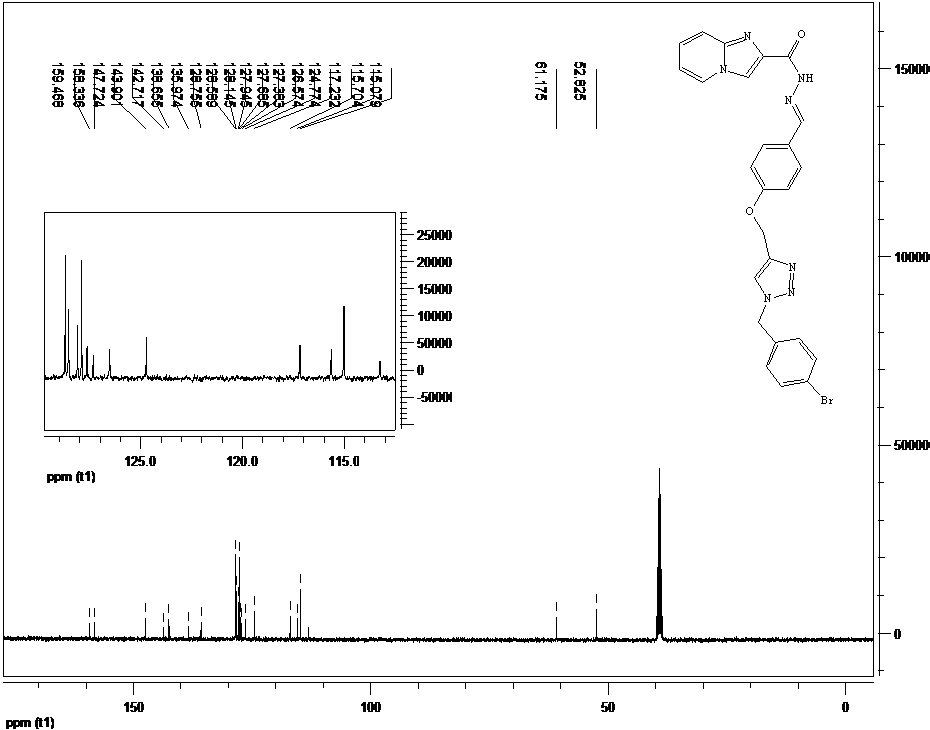


**Fig. S2.** ^13^C NMR spectrum of imidazo [1,2-a]pyridine-2-carboxylic acid {4-[1-(4-bromo-benzyl)-1H-[1,2,3]triazol-4-ylmethoxy]-benzylidene}-hydrazide **(6a)**


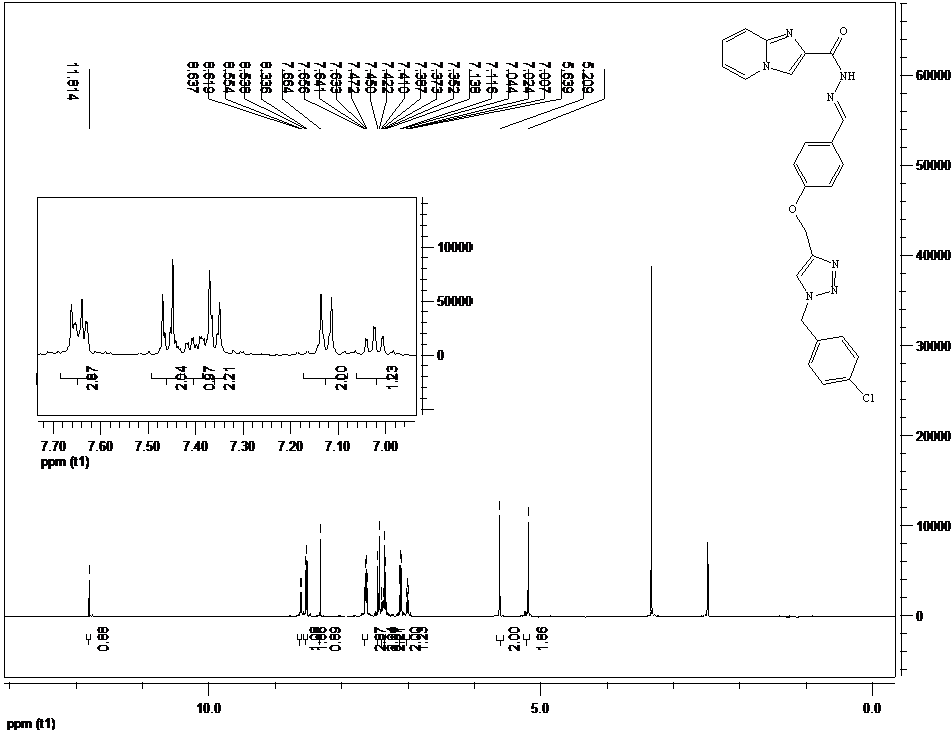


**Fig. S3.** ^1^H NMR spectrum of imidazo [1,2-a]pyridine-2-carboxylic acid {4-[1-(4-chloro-benzyl)-1H-[1,2,3]triazol-4-ylmethoxy]-benzylidene}-hydrazide **(6b)**


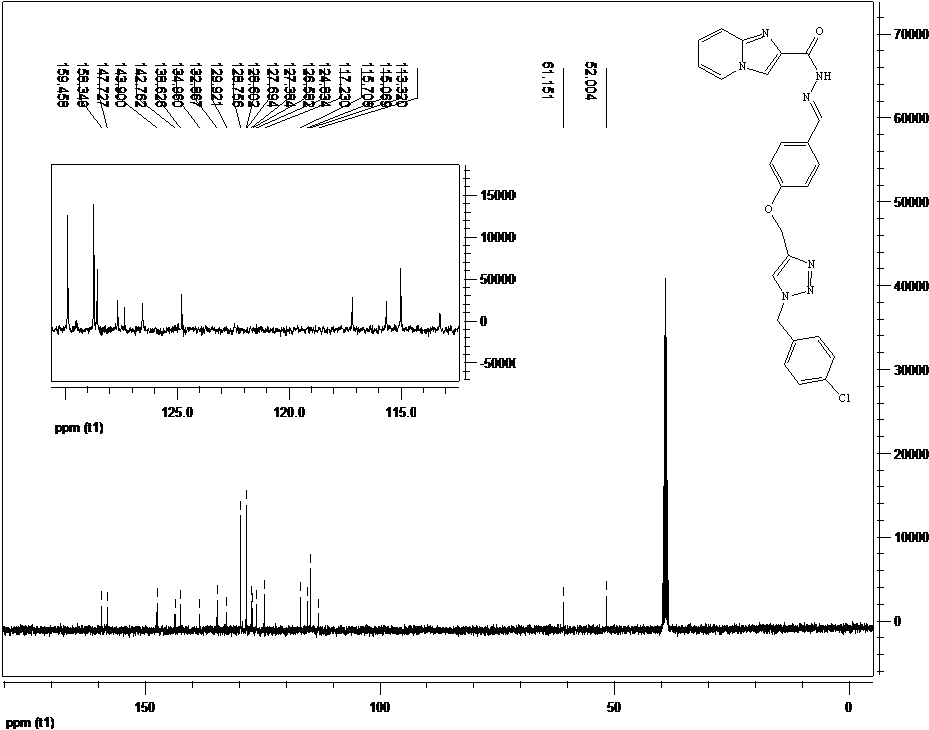


**Fig. S4.** ^13^C NMR spectrum of imidazo [1,2-a]pyridine-2-carboxylic acid {4-[1-(4-chloro-benzyl)-1H-[1,2,3]triazol-4-ylmethoxy]-benzylidene}-hydrazide **(6b)**


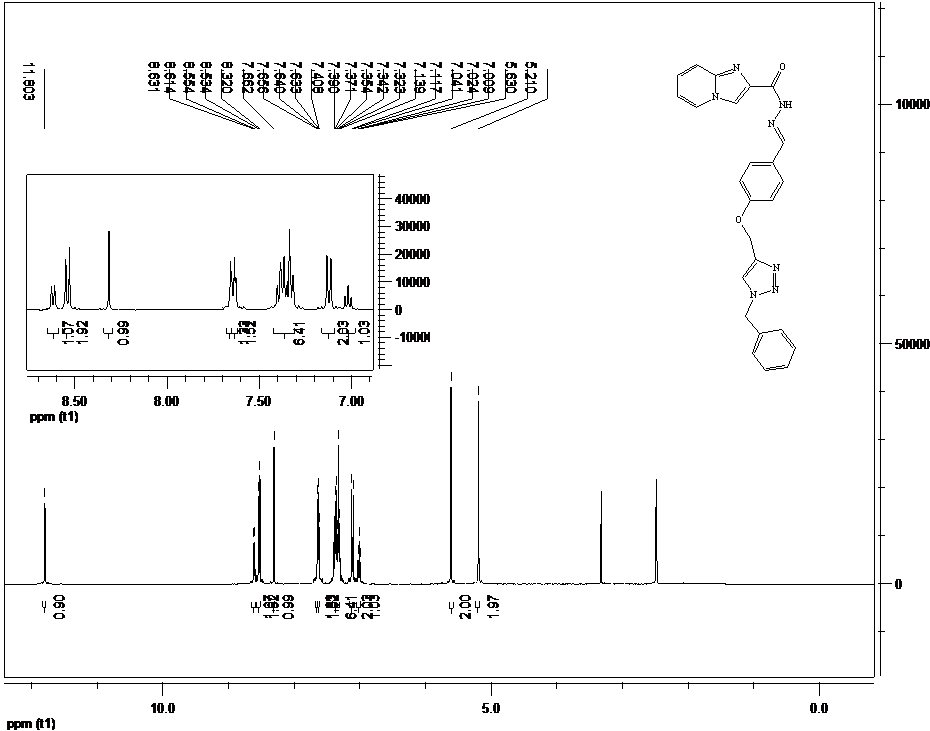


**Fig. S5.** ^1^H NMR spectrum of imidazo [1,2-a]pyridine-2-carboxylic acid [4-(1-benzyl-1H-[1,2,3]triazol-4-ylmethoxy)-benzylidene]-hydrazide **(6c)**


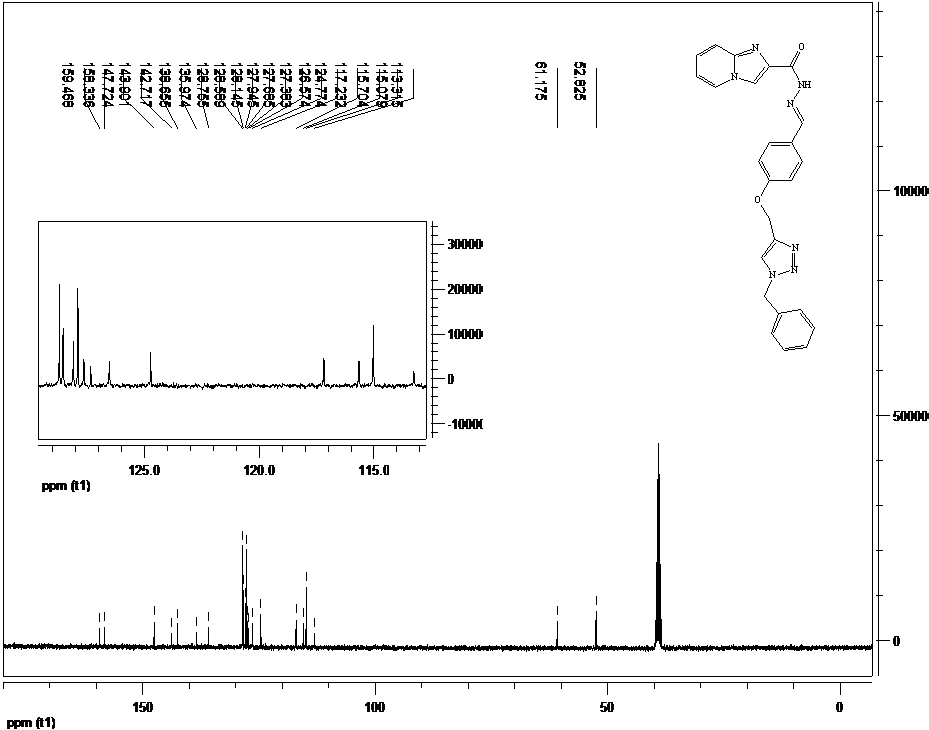


**Fig. S6.** ^13^C NMR spectrum of imidazo 1,2-a]pyridine-2-carboxylic acid [4-(1-benzyl-1H-[1,2,3]triazol-4-ylmethoxy)-benzylidene]-hydrazide **(6c)**


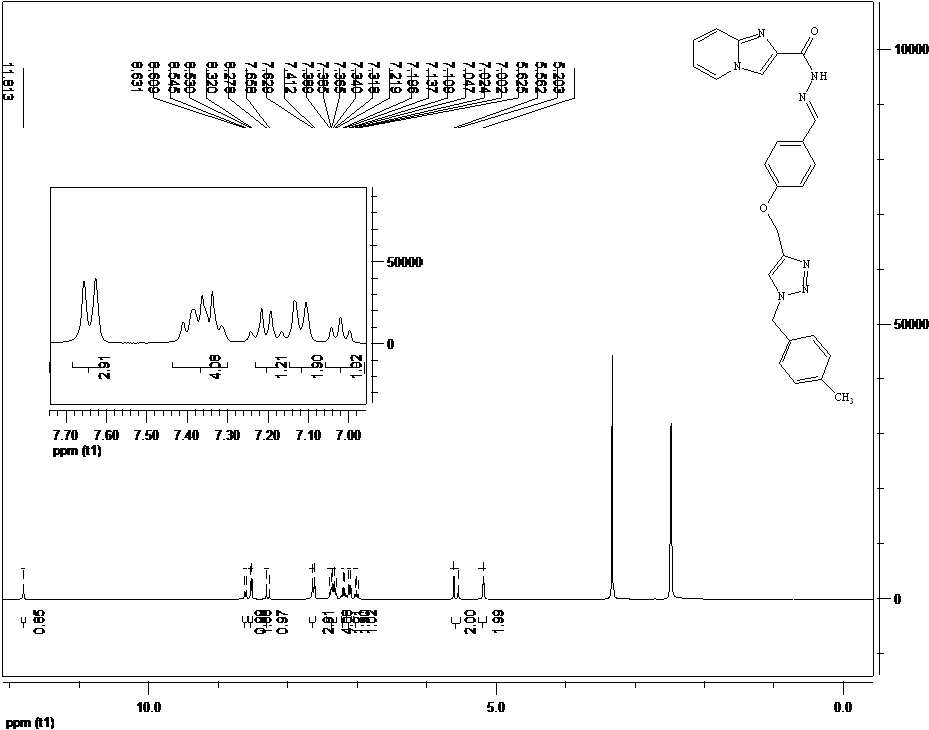


**Fig. S7.** ^1^H NMR spectrum of imidazo [1,2-a]pyridine-2-carboxylic acid {4-[1-(4-methyl-benzyl)-1H-[1,2,3]triazol-4-ylmethoxy]-benzylidene}-hydrazide **(6d)**


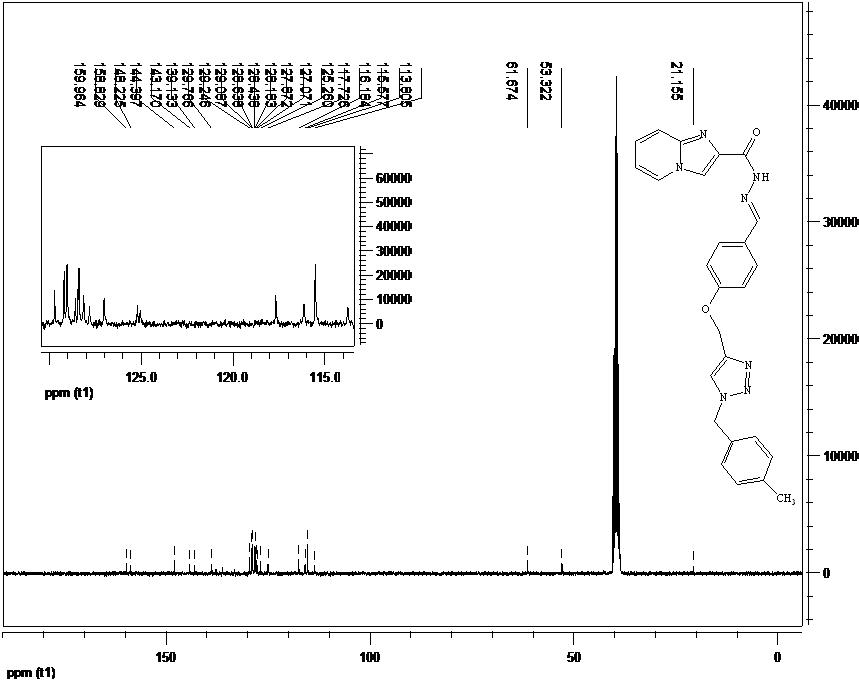


**Fig. S8.** ^13^C NMR spectrum of imidazo [1,2-a]pyridine-2-carboxylic acid {4-[1-(4-methyl-benzyl)-1H-[1,2,3]triazol-4-ylmethoxy]-benzylidene}-hydrazide **(6d)**


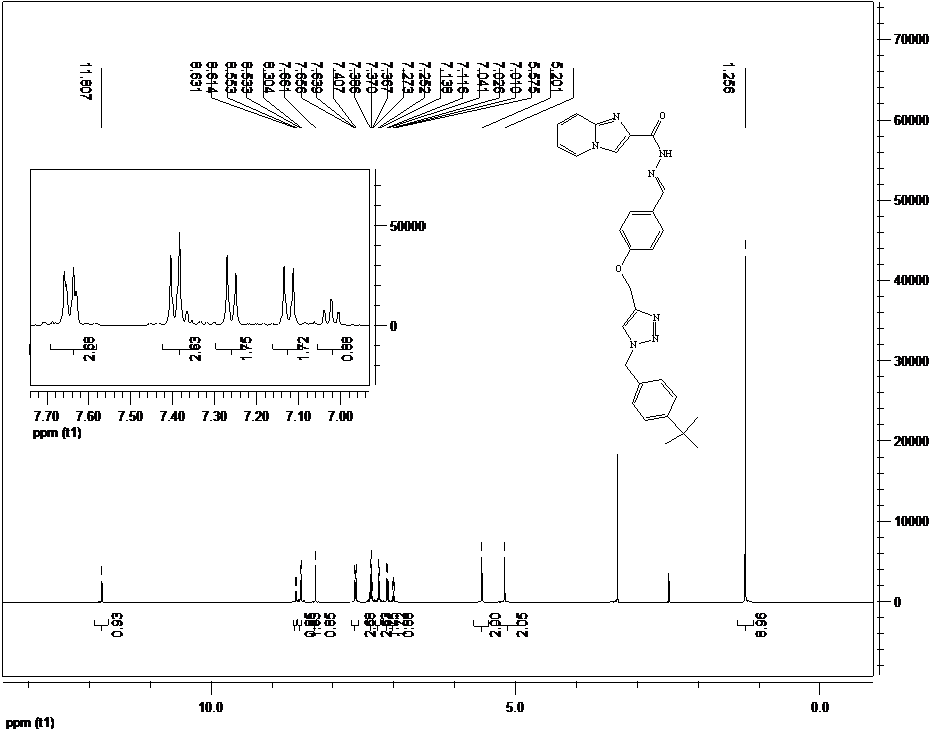


**Fig. S9.** ^1^H NMR spectrum of imidazo [1,2-a]pyridine-2-carboxylic acid {4-[1-(4-tert-butyl-benzyl)-1H-[1,2,3]triazol-4-ylmethoxy]-benzylidene}-hydrazide **(6e)**


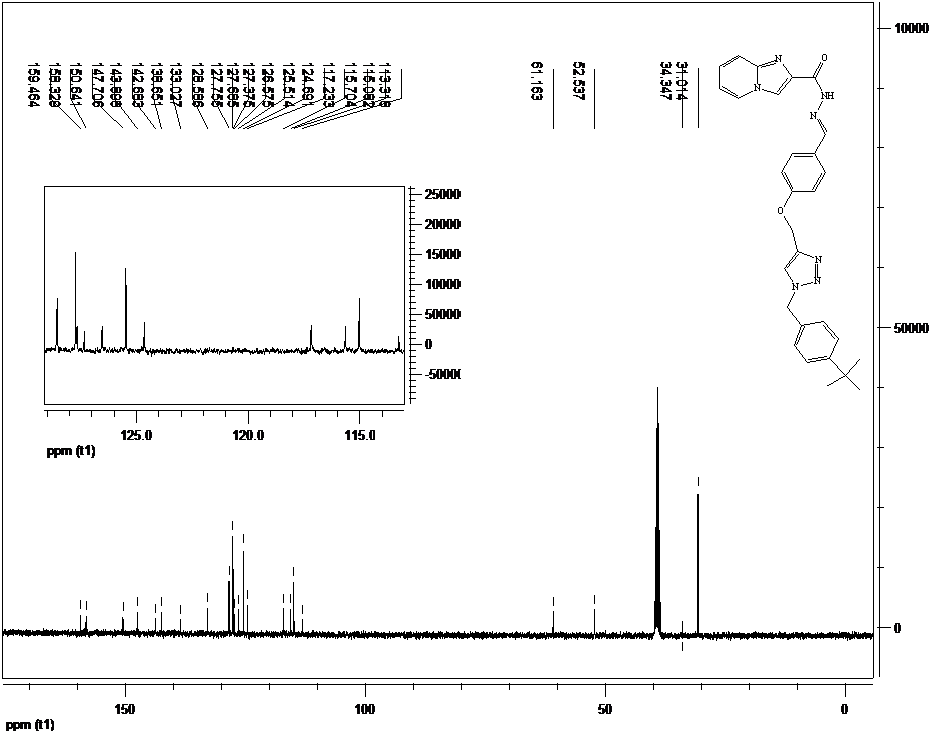


**Fig. S10.** ^13^C NMR spectrum of imidazo [1,2-a]pyridine-2-carboxylic acid {4-[1-(4-tert-butyl-benzyl)-1H-[1,2,3]triazol-4-ylmethoxy]-benzylidene}-hydrazide **(6e)**


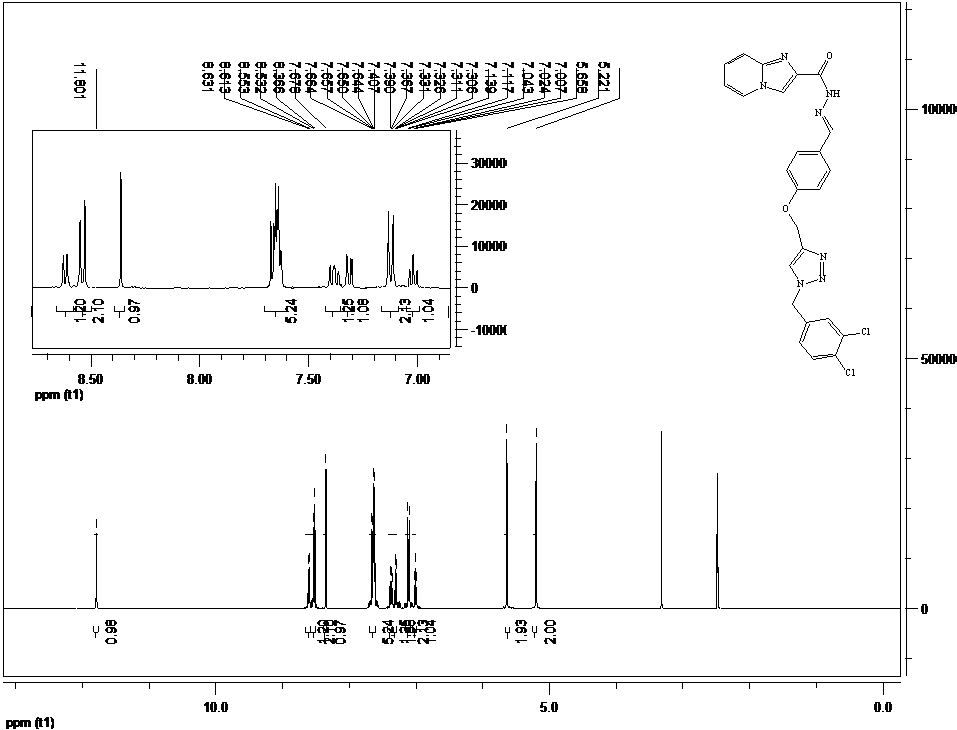


**Fig. S11.** ^1^H NMR spectrum of imidazo [1,2-a]pyridine-2-carboxylic acid {4-[1-(3,4-dichloro-benzyl)-1H-[1,2,3]triazol-4-ylmethoxy]-benzylidene}-hydrazide **(6f)**


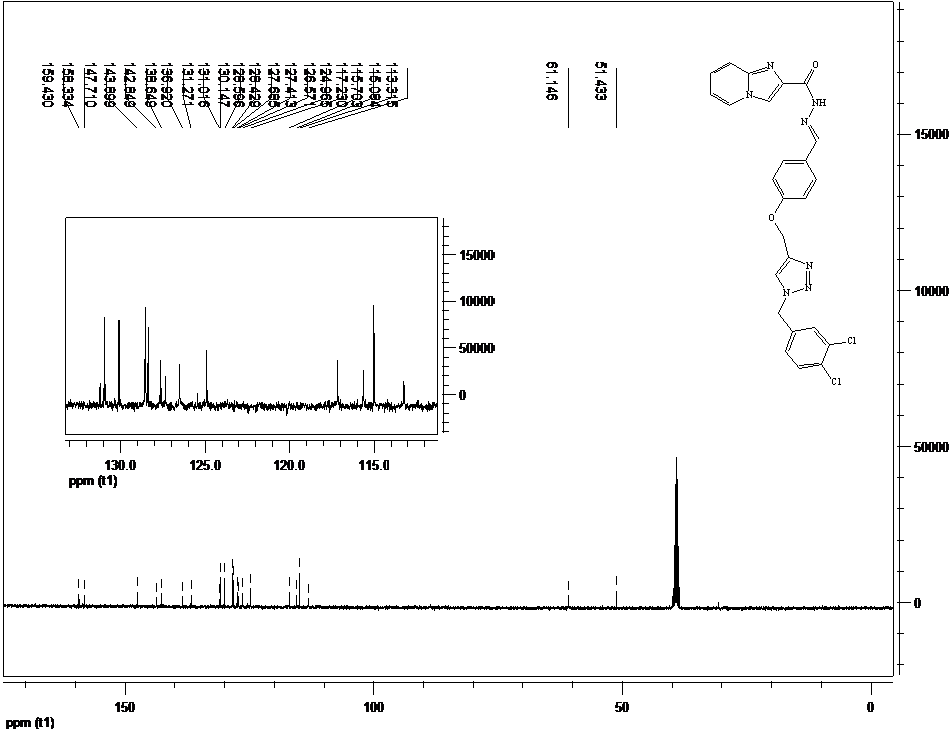


**Fig. S12.** ^13^C NMR spectrum of imidazo [1,2-a]pyridine-2-carboxylic acid {4-[1-(3,4-dichloro-benzyl)-1H-[1,2,3]triazol-4-ylmethoxy]-benzylidene}-hydrazide **(6f)**


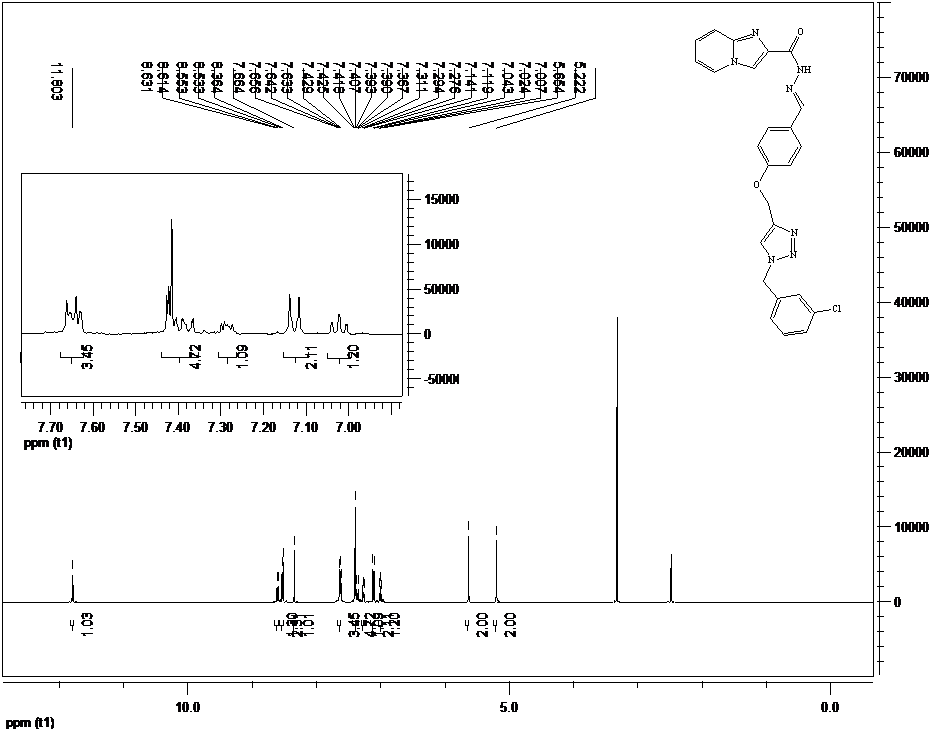


**Fig. S13.** ^1^H NMR spectrum of imidazo [1,2-a]pyridine-2-carboxylic acid {4-[1-(3-chloro-benzyl)-1H-[1,2,3]triazol-4-ylmethoxy]-benzylidene}-hydrazide **(6g)**


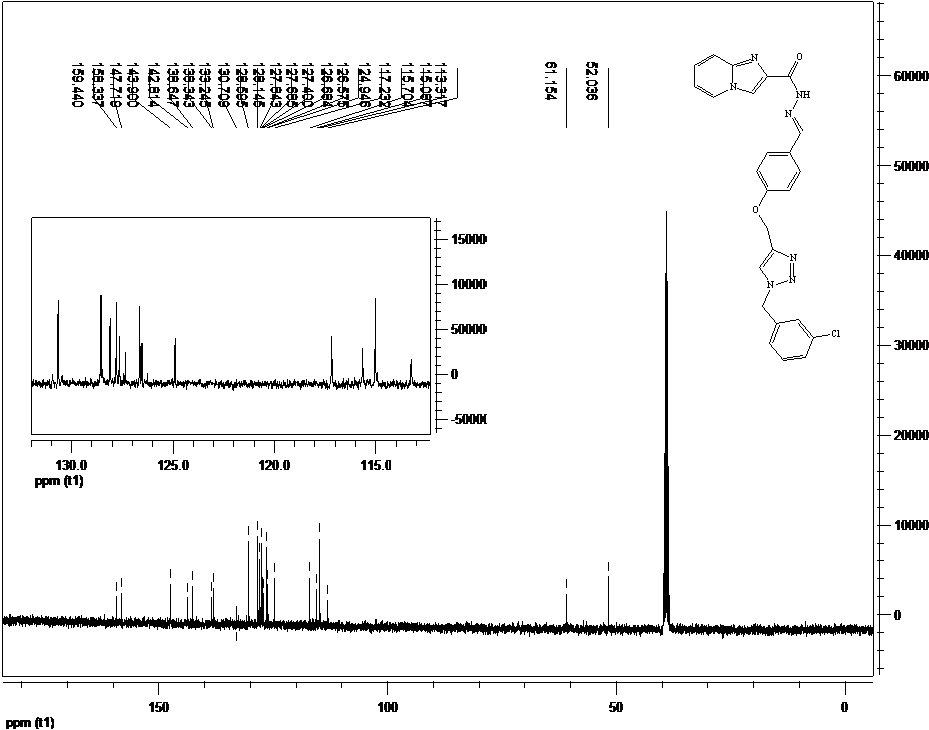


**Fig. S14.** ^13^C NMR spectrum of imidazo [1,2-a]pyridine-2-carboxylic acid {4-[1-(3-chloro-benzyl)-1H-[1,2,3]triazol-4-ylmethoxy]-benzylidene}-hydrazide **(6g)**


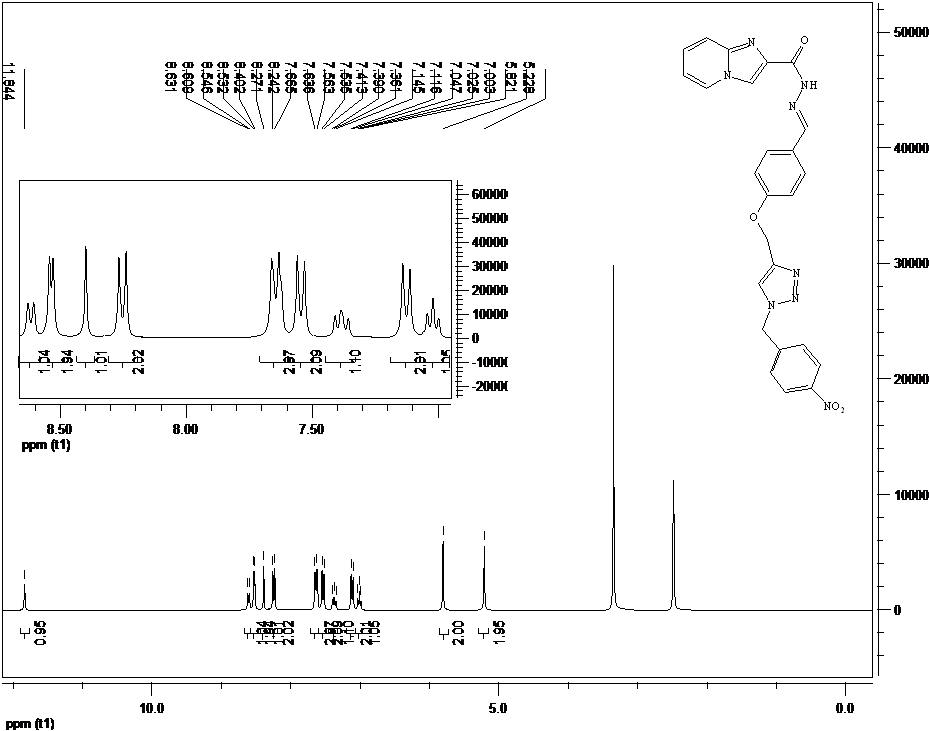


**Fig. S15.** ^1^H NMR spectrum of imidazo [1,2-a]pyridine-2-carboxylic acid {4-[1-(4-nitro-benzyl)-1H-[1,2,3]triazol-4-ylmethoxy]-benzylidene}-hydrazide **(6h)**


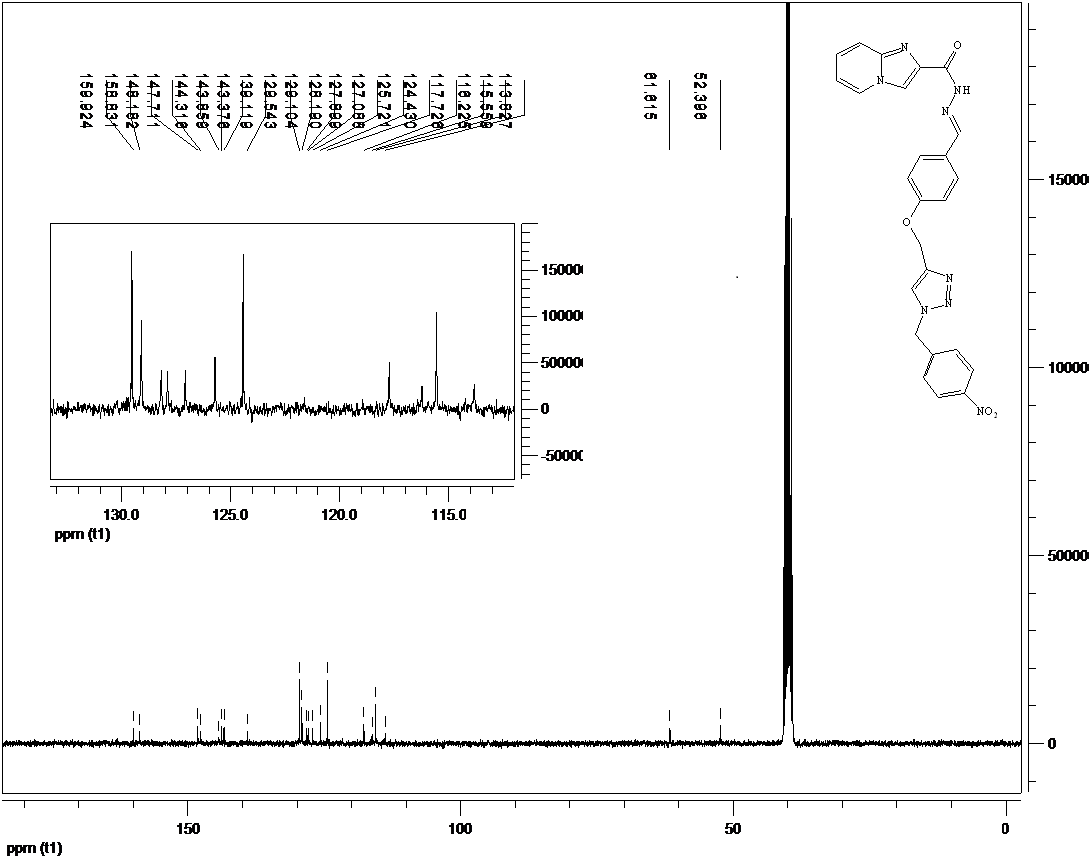


**Fig. S16.** ^13^C NMR spectrum of imidazo [1,2-a]pyridine-2-carboxylic acid {4-[1-(4-nitro-benzyl)-1H-[1,2,3]triazol-4-ylmethoxy]-benzylidene}-hydrazide **(6h)**


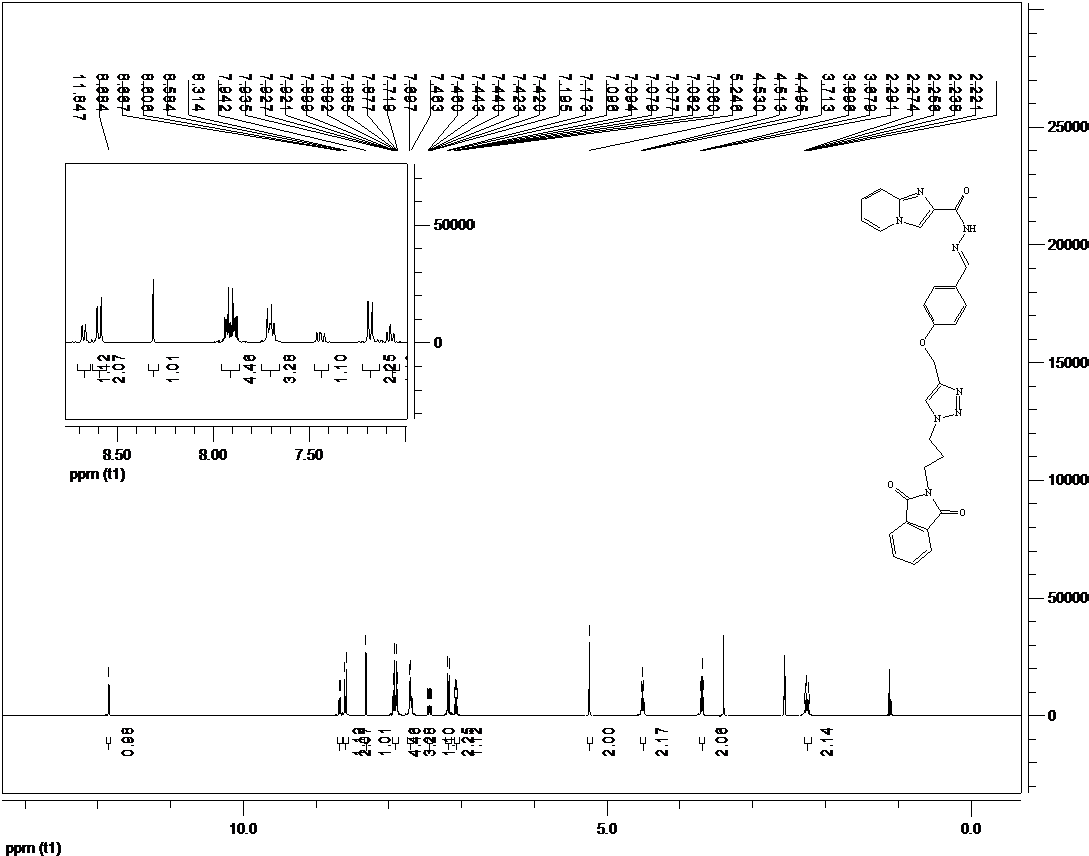


**Fig. S17.** ^1^H NMR spectrum of imidazo [1,2-a]pyridine-2-carboxylic acid (4-{1-[3-(1,3-dioxo-1,3-dihydro-isoindol-2-yl)-propyl]-1H-[1,2,3]triazol-4-ylmethoxy}-benzylidene)-hydrazide **(6i)**


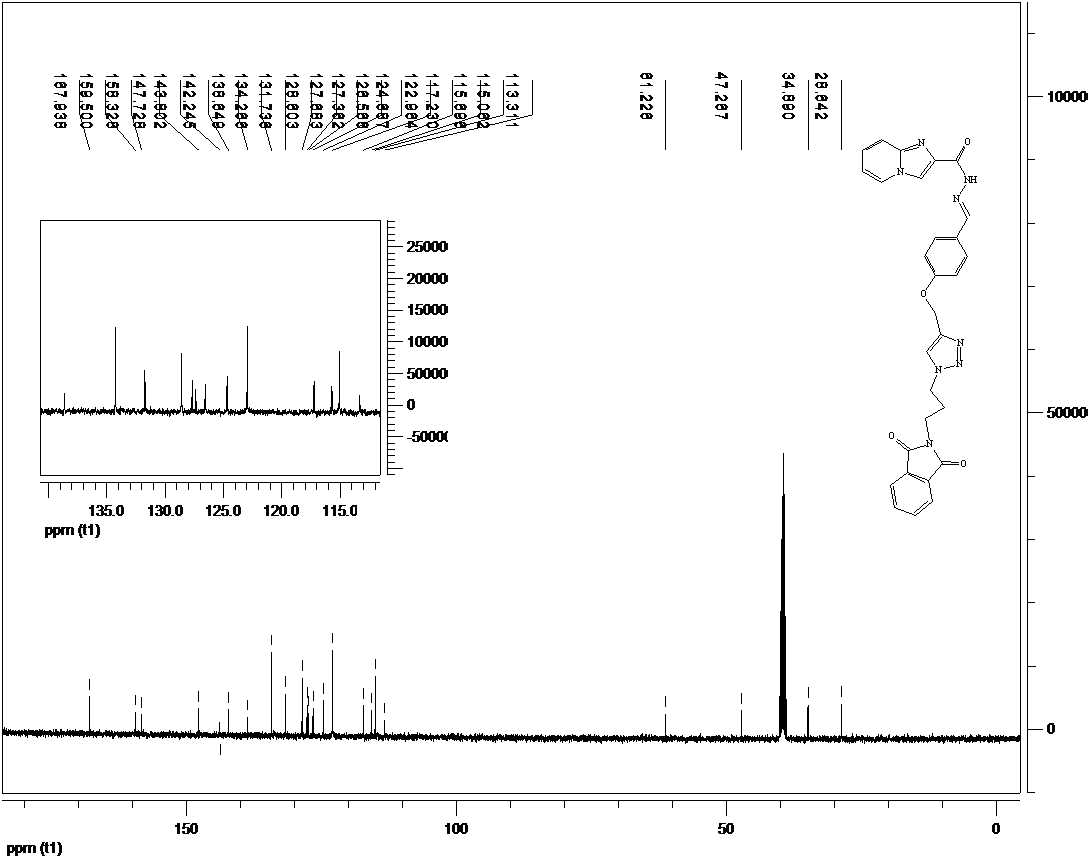


**Fig. S18.** ^13^C NMR spectrum of imidazo [1,2-a]pyridine-2-carboxylic acid (4-{1-[3-(1,3-dioxo-1,3-dihydro-isoindol-2-yl)-propyl]-1H-[1,2,3]triazol-4-ylmethoxy}-benzylidene)-hydrazide **(6i)**


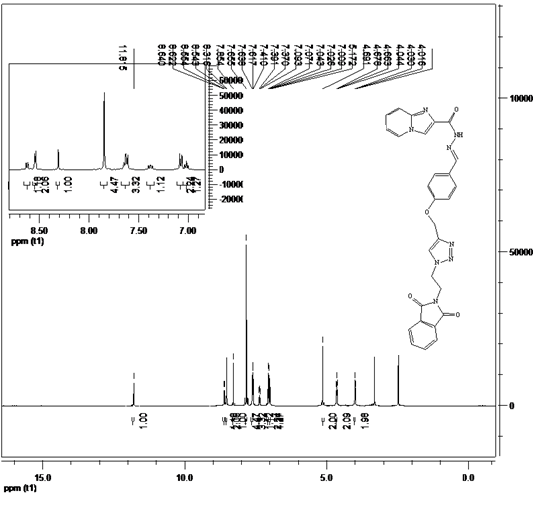


**Fig. S19.** ^1^H NMR spectrum of imidazo [1,2-a]pyridine-2-carboxylic acid (4-{1-[2-(1,3-dioxo-1,3-dihydro-isoindol-2-yl)-ethyl]-1H-[1,2,3]triazol-4-ylmethoxy}-benzylidene)-hydrazide **(6j)**


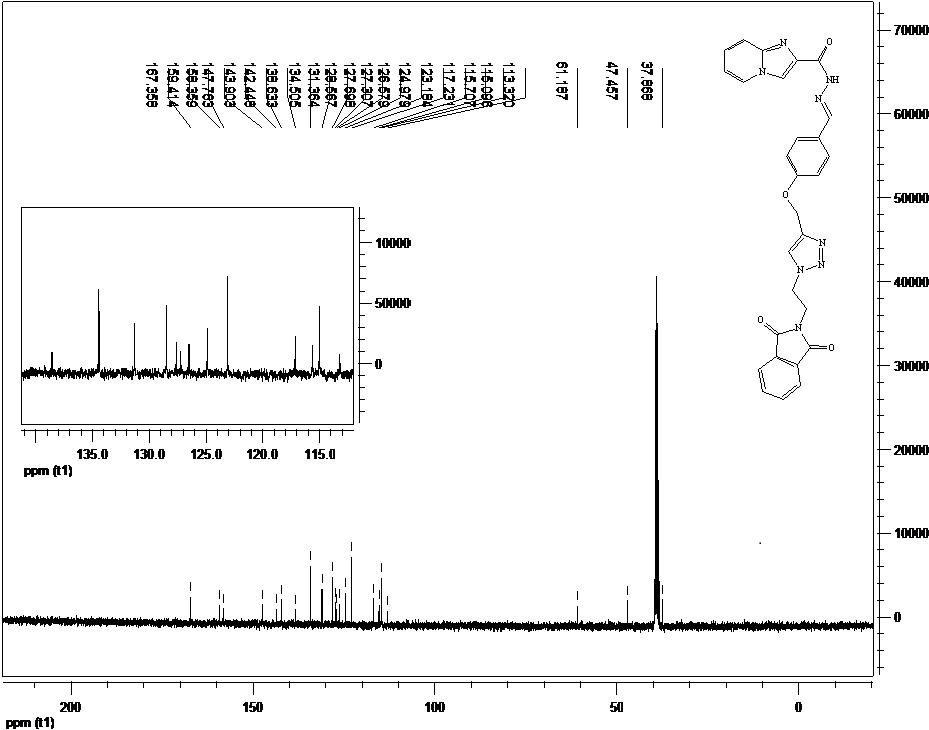


**Fig. S20.** ^13^C NMR spectrum of imidazo [1,2-a]pyridine-2-carboxylic acid (4-{1-[2-(1,3-dioxo-1,3-dihydro-isoindol-2-yl)-ethyl]-1H-[1,2,3]triazol-4-ylmethoxy}-benzylidene)-hydrazide **(6j)**


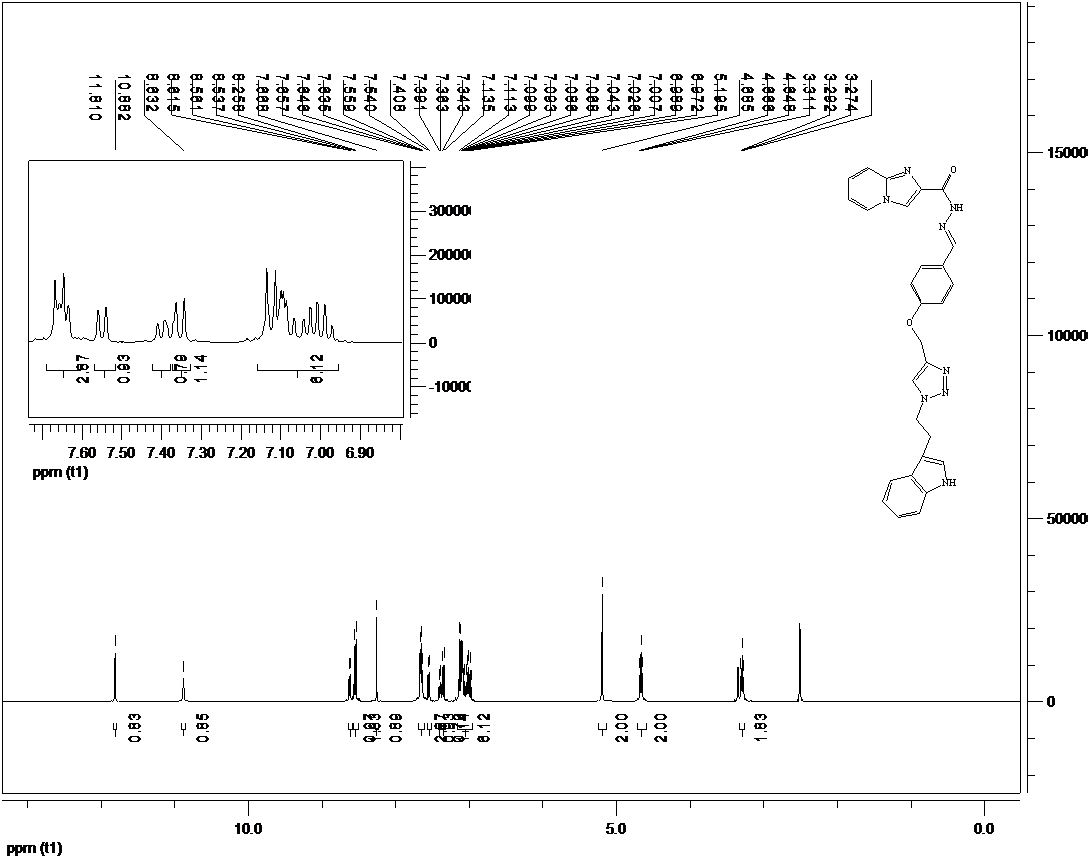


**Fig. S21.** ^1^H NMR spectrum of imidazo [1,2-a]pyridine-2-carboxylic acid (4-{1-[2-(1H-indol-3-yl)-ethyl]-1H-[1,2,3]triazol-4-ylmethoxy}-benzylidene)-hydrazide **(6k)**


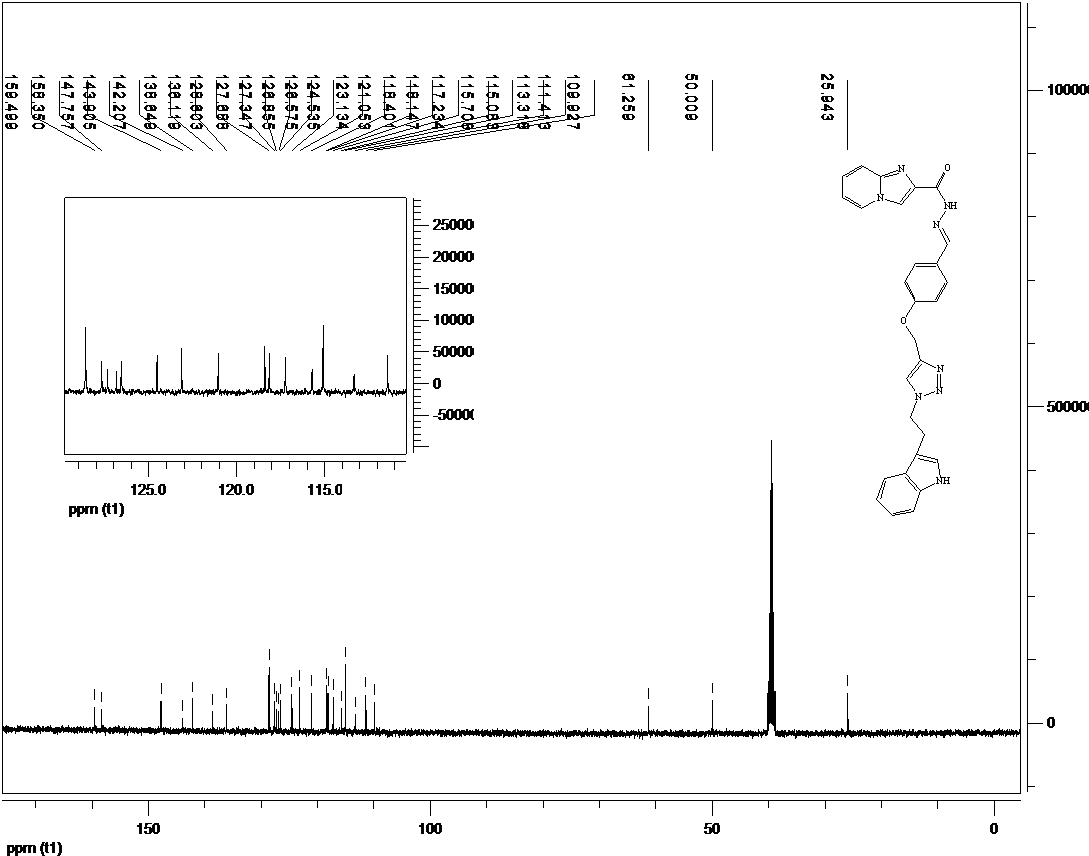


**Fig. S22.** ^13^C NMR spectrum of imidazo imidazo [1,2-a]pyridine-2-carboxylic acid (4-{1-[2-(1H-indol-3-yl)-ethyl]-1H-[1,2,3]triazol-4-ylmethoxy}-benzylidene)-hydrazide **(6k)**


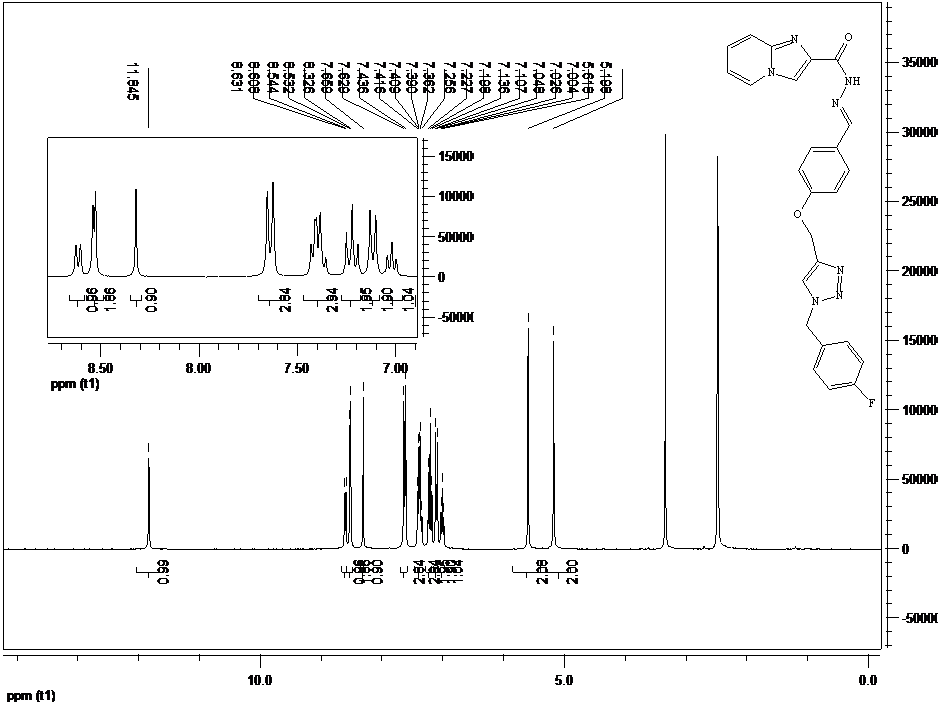


**Fig. S23.** ^1^H NMR spectrum of imidazo [1,2-a]pyridine-2-carboxylic acid {4-[1-(4-fluoro-benzyl)-1H-[1,2,3]triazol-4-ylmethoxy]-benzylidene}-hydrazide **(6l)**


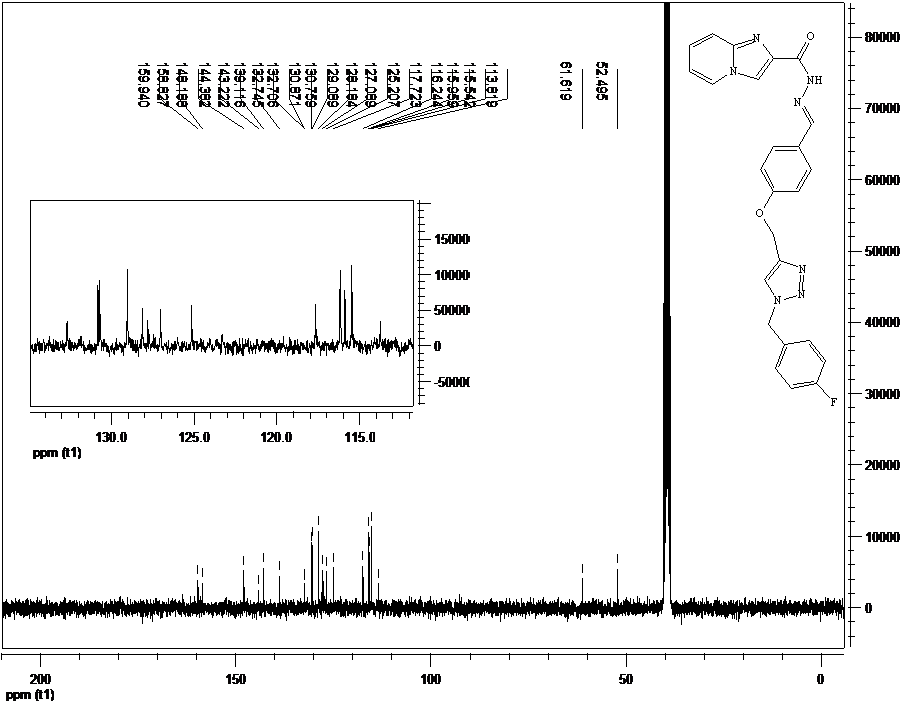


**Fig. S24.** ^13^C NMR spectrum of imidazo [1,2-a]pyridine-2-carboxylic acid {4-[1-(4-fluoro-benzyl)-1H-[1,2,3]triazol-4-ylmethoxy]-benzylidene}-hydrazide **(6l)**

| **Ligand** | **Average binding energy** | **Ligand binding energy contribution** | |
| --- | --- | --- | --- |
| Foretinib | -139.385±16.428 | -66.562 | |
| LY2801653 | -207.329 ± 15.932 | | -109.369 |
| 75H | -198.225±14.013 | -92.29 | |
| 66L | -199.046±14.443 | -98.48 | |
| 6d | -176.661 ± 14.073 | -89.662 | |
| 6e | -168.035 ± 13.897 | -102.271 | |
| 6f | -165.792 ± 17.632 | -79.360 | |

**Table.S1**. Average binding energy and energy contribution of ligands inside the active site of c-Met obtained from MM-PBSA analysis in kJ/mol.
