# Supplementary material for: Imidazopyridine hydrazone derivatives exert antiproliferative effect on lung and pancreatic cancer cells and potentially inhibit receptor tyrosine kinases including c-Met
Source: Sci Rep. 2021 Feb 11;11:3644. doi: 10.1038/s41598-021-83069-4 (PMC7878917; doi:10.1038/s41598-021-83069-4)
Supplement: Supplementary file 2 — Supplementary Information 2. [file 41598_2021_83069_MOESM2_ESM.docx]

| **A**  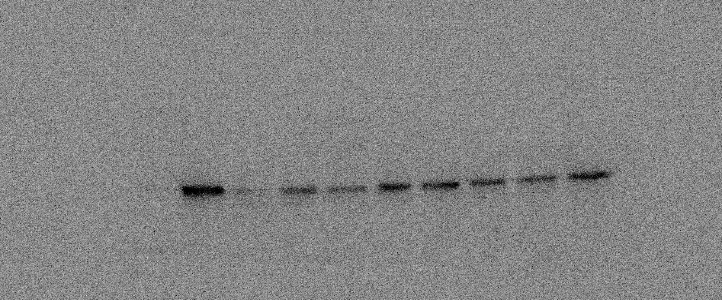 |
| --- |
| **B**  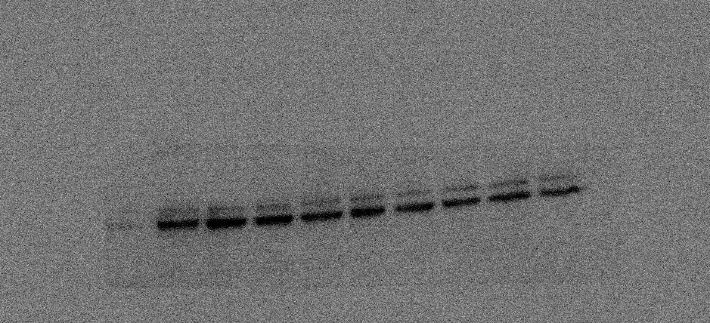 |

**Supplementary information 1.** **Raw pictures of the western blots presented in Figure 4.** Effect of synthesized derivatives on the phospho*-c-*Met (A) *and*total c-Met protein expression (B) are shown.
